# Spatial transcriptomics analysis uncovers ER stress in MANF-deficient Purkinje cells underlying alcohol-induced cerebellar neurodegeneration in mice

**DOI:** 10.1186/s40478-025-02162-1

**Published:** 2025-12-03

**Authors:** Wen Wen, Hui Li, Li-Chun Lin, Michael S. Chimenti, Henry L. Keen, Mariah R. Leidinger, Di Hu, Zuohui Zhang, Hong Lin, Jia Luo

**Affiliations:** 1 Department of Pathology, Carver College of Medicine, University of Iowa, Iowa City, IA 52242 USA; 2 Iowa Neuroscience Institute, Carver College of Medicine, University of Iowa, Iowa City, IA 52242 USA; 3 Bioinformatics Division, Iowa Institute of Human Genetics, Carver College of Medicine, University of Iowa, Iowa City, IA 52242 USA; 4 Comparative Pathology Laboratory, Carver College of Medicine, University of Iowa, Iowa City, IA 52242 USA; 5 Iowa City VA Health Care System, Iowa City, IA 52246 USA

**Keywords:** Alcohol use disorder, Cerebellar dysfunction, Sex difference, Spatial transcriptomics, Unfolded protein response

## Abstract

Alcohol use disorders (AUD) is one of the most prevalent mental disorders in the United States affecting more than 10% of the adult population. Cerebellar atrophy and Purkinje cell (PC) degeneration are frequently observed in patients with AUD. Alcohol can cause endoplasmic reticulum (ER) stress in PCs and alter PC structure and function. Mesencephalic astrocyte-derived neurotrophic factor (MANF) is an ER stress inducible protein highly expressed in PCs. It is neuroprotective in various ER stress-related pathological conditions. However, it is unknown whether MANF plays a role in protecting PCs from alcohol-induced ER stress and neurodegeneration. In this study, we generated PC-specific MANF knockout (KO) mouse model to test the hypothesis that MANF-deficient PCs are more susceptible to alcohol-induced ER stress and neurodegeneration in the adult brain. We employed a binge alcohol exposure paradigm and tested the effect of alcohol and MANF deficiency on molecular, cellular, and behavioral outcomes in the adult animals. We also performed spatial transcriptomics and high throughput in situ analyses to profile gene expression changes in response to MANF deficiency. We found that alcohol exacerbated the motor function deficits in PC-specific MANF KO animals. Interestingly, female KOs were more sensitive to alcohol-induced motor function impairments than male KOs. In accordance with the behavior changes, alcohol exposure activated the unfolded protein response (UPR), increased intranuclear expression of calcium binding protein, and caused PC degeneration in female but not male MANF KO mice. Spatial transcriptomics and high throughput Xenium in situ analyses revealed that MANF deficiency altered the transcriptomic landscape in PCs in a sex-specific manner and triggered the expression of genes involved in protein folding and transportation, and response to ER stress. Our study reveals that MANF-deficient PCs are predisposed with a higher risk to UPR activation and disrupted calcium homeostasis in a sex-dependent manner, which may underline their sex-specific vulnerability to alcohol-induced neurodegeneration. These findings suggest that ER stress plays a significant role in alcohol-triggered neurodegenerative process in the cerebellum, and MANF may possess therapeutic potentials in AUD via its capacity in restoring ER and calcium homeostasis.

**Supplementary Information:**

The online version contains supplementary material available at 10.1186/s40478-025-02162-1.

## Introduction

Excessive alcohol consumption has profound adverse effects on the brain, affecting both neuronal structure and function. The cerebellum is one of the most vulnerable brain regions to the toxic effects of alcohol [69]. Even low levels of acute alcohol intoxication can impair cerebellar function, leading to noticeable motor deficits such as unsteady gait and poor coordination [20]. Chronic alcohol consumption and binge drinking can lead to irreversible cerebellar degeneration, which is one of the most common neurological complications in patients with alcohol use disorder (AUD). The cerebellum is not only responsible for coordinating motor movements but also involves in non-motor functions such as memory, language, and emotional control. As a result, alcohol-induced cerebellar degeneration can lead to long-term motor impairments and may also play a significant role in the adverse impact of alcohol on cognition and emotion [26]. Excessive alcohol exposure is associated with structural abnormalities and neurologic cerebellar dysfunction with anterior and superior vermis atrophy, decrease in the volume of molecular and granular layers, and loss of Purkinje cells (PCs) [73, 86]. PCs are the sole efferent neurons of the cerebellum, sending output from the cerebellar cortex to the cerebral cortex and brain stem. Alcohol exposure during early development or chronic alcohol exposure in adult can alter the structure and function of PCs [21–23, 27, 36, 82, 99, 116]. Cerebellar atrophy and PC degeneration are often reported in postmortem human brains in individuals with chronic alcohol consumption, with some studies showing a loss of PCs up to 40% [5, 9, 10, 48, 81, 96, 125].

The mechanisms by which alcohol causes PC degeneration are complex and multifaceted, involving direct neurotoxic effects of ethanol and its metabolic products, nutritional deficiencies, such as thiamine deficiency, potential immune-mediated processes, and dysregulation of neurotrophic factors [73]. Animal studies indicate that alcohol cause PC shrinkage, dendritic arbor reduction, mitochondrial fragmentation, disruption in Ca^2+^ signaling, synaptic impairment, electrophysiological changes, and neurotransmission alteration [14, 22, 27, 33, 51, 78, 99]. Understanding the impact of alcohol on the cerebellum is crucial for developing effective interventions and treatments for alcohol-related brain damages. Alcohol-induced endoplasmic reticulum (ER) stress is associated with multiple organ damages [45]. We and other investigators have demonstrated that alcohol exposure induces ER stress in both the developing and mature brain, including the cerebellum [21, 31, 32, 50, 61, 112–114, 117, 118, 121]. The ER is an important organelle for the biological function and homeostasis of cells, regulating posttranslational modification, folding, and transportation of proteins, and the synthesis of membrane lipids and cholesterol. ER chaperone molecules such as the 78-kDa glucose-regulated protein (GRP78) facilitates protein folding to maintain ER protein homeostasis in non-stressed condition. Under stress conditions such as pathogen infection, nutrient deprivation, and inflammation, the abundance of misfolded proteins access the capacity of the protein folding machinery in the ER, the unfolded protein response (UPR) will be triggered via the activation of three ER transmembrane receptors: pancreatic ER kinase-like ER kinase (PERK), inositol-requiring enzyme 1α (IRE1α), and activating transcription factor 6 (ATF6) [104]. UPR alleviates ER stress by suppressing the rate of protein synthesis, degrading the unfolded or misfolded proteins through ER-associated protein degradation (ERAD), and producing molecular chaperons to increase the protein folding capacity in the ER [104]. If the UPR is insufficient to overcome ER stress, prolonged ER stress can ultimately results in apoptosis [84]. Chronic alcohol exposure has been reported to increase the expression of ER stress markers in the PCs and induce dilation of the smooth endoplasmic reticulum (SER) which precedes and accompanies dendritic regression of PCs in rats [21–23].

Mesencephalic astrocyte-derived neurotrophic factor (MANF) is an evolutionarily conserved ER resident protein belonging to a novel neurotrophic factor family [19]. It was originally identified as a secreted trophic factor for dopamine neurons in vitro [80]. It is systemically expressed throughout the nerves and non-nervous systems. In the rodent brain, robust MANF expression can be detected in neurons from embryonic stages to adulthood and declined during aging, suggesting its role in neurodevelopment and maintenance of neuronal functions [65, 93, 109]. MANF has a cytoprotective role in regulating UPR and maintaining ER homeostasis [8, 67]. MANF protein physically interacts with the major ER chaperone GRP78 [120]. Constitutive MANF-deficient mouse exhibit chronic UPR activation in the brain [98]. MANF expression and secretion are upregulated in response to ER stress [7, 77, 110]. Generally, MANF upregulation offers protection from ER stress in experimental models of Parkinson’s disease, brain and heart ischemia, and retinal degeneration [3, 28, 29, 35, 65, 103, 126]. Several MANF binding proteins were identified both inside the cell and on the cell surface that may mediate its trophic functions. Inside the cell, MANF binds to the ER membrane receptor IRE1α that mediates the UPR and promotes neuronal survival [55]. On the cell surface, MANF can bind to the transmembrane protein Neuroplastin (NPTN) to promote cellular survival and regulate NF-κB associated inflammation [119]. Conventional MANF knockout mice develop diabetes, hearing loss, liver defects, and impaired neural migration and neurite outgrowth, with activated UPR [63, 97]. Patients lacking functional MANF also exhibit diabetes and hearing loss [75, 124].

Previously, we have demonstrated that acute alcohol exposure caused significant upregulation of MANF in the developing mice brain which was accompanied with increased ER stress [50, 61]. MANF is highly expressed in PCs in the adult mouse brain [65]. It has been reported to protect and restore damaged PCs in mouse model of spinocerebellar ataxia (SCA) [122]. However, whether MANF protects PCs from alcohol-induced degeneration is unknown. In the present study, we generated a PC-specific MANF KO mouse model to test the hypothesis that MANF-deficient PCs are more susceptible to alcohol-induced ER stress and neurodegeneration. Furthermore, we examined the transcriptomic changes due to MANF deficiency in PCs to identify genes and signaling pathways that potentially interacted with MANF using Visium spatial transcriptomics analysis (STA) followed by Xenium in situ analysis. We identified baseline gene expression changes caused by MANF loss, which may underlie the increased vulnerability of PCs to alcohol neurotoxicity.

## Materials and methods

### Animal husbandry

All animals were group housed (up to 5 per cage) and cared for by the Office of Animal Resources at the University of Iowa. Mice were allowed ad libitum access to chow and water on a 12 h light/12 h dark cycle. All experimental animal procedures were approved by the Institutional Animal Care and Use Committee at the University of Iowa (#3042295) and performed following regulations for the Care and Use of Laboratory Animals set forth by the National Institutes of Health Guide.

### Mouse strains and genotyping

Adult male and female mice were used for this study. *Manf* ^fl^/^fl^ transgenic mice with C57BL/6 background were generated as described previously [113, 114]. Purkinje cell specific Cre mice B6.Cg-Tg(Pcp2-cre)3555Jdhu/J (referred to as *Pcp2-Cre* and thereafter) were purchased from The Jackson Laboratory. *Manf* ^fl/fl^ mice and *Pcp2-Cre*^+/−^ mice were crossed for two generations to generate *Manf* ^fl/fl^; *Pcp2-Cre*^+/−^ mice which have specific MANF knockout in the PCs (referred to as KO and thereafter). Their littermates that were *Manf* ^fl/fl^; *Pcp2-Cre*^−/−^ were used as control animals (referred to as control and thereafter). All mice were genotyped by polymerase chain reaction (PCR) analysis using the Fast Tissue/Tail PCR Genotyping Kit (G1001, EZ BioResearch) with primers (Integrated DNA Technologies) as follows: Manf (forward) 5’- TGAAGCAAGAGGCAAAGAGAATCGG-3’, Manf (reverse) 5’- TGCTCAGCTGCAGAGTTAGAGTTCC-3’; Cre (forward) 5’-GGTTCGCAAGAACCTGATGG-3’, Cre (reverse) 5’-GCCTTCTCTACACCTGCGG-3’. PCR products were visualized by electrophoresis on 1% agarose gel and stained with ethidium bromide (Thermo Fisher Scientific).

### Alcohol administration

A binge alcohol exposure paradigm was used for this study as it has been widely used to produce alcohol neurotoxicity in rodents, mimicking the neuropathological conditions in human with alcohol use disorders [47]. Both male and female adult mice at the age of four to five months old received equal volume of H_2_O or ethanol (200 proof, Decon Labs, Inc, Swedeland, PA) (5 g/kg, 25% ethanol w/v) via intragastric gavage once daily at approximately 2:00 pm for 10 consecutive days. The resulting blood alcohol concentration (BAC) peaks above 300 mg/dl one hour after the last ethanol administration. [112]. This alcohol exposure paradigm corresponds to severe alcohol intoxication in human binge drinkers with impaired motor coordination, sedation, and neurotoxicity. All mice were given ad libitum access to food and water throughout the period of alcohol administration.

### Behavioral tests

Behavioral tests were performed 10 days after the last alcohol exposure to measure persistent neurobehavioral deficits attribute to alcohol neurotoxicity with minimized effect of acute alcohol intoxication and withdrawal. A total of 147 animals (74 males, 73 females) were used for behavior tests, with at least 8 animals in each treatment group for all the tests. Two cohorts of mice were used for behavioral testing. Both cohorts received identical treatments at matched ages and with the same schedule. Cohort 1 was used for the open field, rotarod, and balance beam tests, and Cohort 2 for the 3-chamber sociability test and Barnes maze. Separate cohorts were used to avoid excessive behavioral testing that would have delayed assessment and allowed potential recovery from alcohol-induced molecular changes. Age, sex, and genotype were balanced across all treatment groups. All mice were given ad libitum access to food and water throughout the tests. All behavioral tests were performed during the light phase. Behavioral experiments and subsequent data analyses were carried out by an experimenter blinded to the genotype and treatment group of the animals.

### Open field

The open field test is a common behavioral assay that measures locomotor activity and anxiety-like behaviors in rodents [87]. Before the test, animals were brought to the testing room in a clean holding cage to acclimate for 30 min as a habituation period. The test was conducted 15 min for each animal in a multi-unit open field arena measured 50 cm × 50 cm × 40 cm (San Diego Instruments, San Diego, CA). The center of the open field was defined as the central zone with a length and width half the length and width of the chamber. All testing was conducted under uniform diffuse lighting of 110–130 lx across the chamber. The total distance travelled, and time spent in the center zone were recorded using the EthoVision XT 17 video-tracking software (Noldus Information Technology, Leesburg, VA, USA).

### Rotarod test

The rotarod test is a commonly used method to evaluate the motor coordination deficits in rodents using an animal’s natural avoidance of fall. All testing was conducted under moderate diffuse lighting of ~ 70 lx. Mice were placed on a rotating rod 18″ above the ground in the rotarod apparatus (San Diego Instruments, San Diego, CA). The latency to fall, which is the accumulative time for each mouse to remain on the rod, was recorded automatically with infrared sensors as an indicator for motor coordination. Mice were first trained on the rotating rod at a constant speed of 4 rpm for 60 s. Then the rod was rotated from a speed of 4 rpm to 40 rpm over the course of 300 s. Three consecutive 300 s trials were performed for each mouse, and the latency to fall was recorded, in which 300 s were set as the maximum time. The mean of the 3 trials was calculated for each mouse.

### Balance beam test

Balance beam test was used to assess motor coordination. All testing was conducted under moderate diffuse lighting of ~ 70 lx. Mice were placed at the end of an 80 cm-long wood beam elevated 50 cm from the floor. An open cage was attached to the other end of the beam. During the training phase, each animal was allowed to cross a beam (3.0 cm wide) freely twice. After training, mice were required to cross the beams with various width (2.0 cm, 1.0 cm, and 0.5 cm) from one end to the other end in the open cage. The time spent for the crossing was recorded for each animal. If an animal stopped crossing the beam, its tail was gently touched to encourage movement. If an animal fell from the beam, it was placed back on the end of the beam to restart crossing.

### Three-chamber sociability task

Testing was conducted under indirect low light of 20 lx. At least 30 min before testing starts, all animals were acclimated in the testing room. A matte black plastic box arena with three chambers and openings between the chambers was custom made for the test. The left- and right-side chambers measured 10″ × 7.5″ × 7.5″ (length x width x height). They each contains a transparent plastic cylinder to enclose the stimulus mouse/object. The cylinders were 2.75″ in diameter and 4.75″ in height with 0.375″ diameter circular holes to allow olfactory, auditory, and some tactile cues while preventing direct physical contact. Each animal was habituated in the arena with empty cylinders for 10 min. Then it is followed with 10 min testing session, during which a novel mouse was placed in one chamber’s cylinder and a novel object (a piece of brightly yellow colored toy Mega Blok, approximately 1.25″ × 1.25″ × 2.75″ length x width x height) was placed in the other chamber’s cylinder. Novel mice were wild type C57BL/6 mice of the same sex and age that the tested mice had never been exposed to previously. The total distance travelled, and the time spent in each chamber and with each cylinder were recorded and analyzed by the EthoVision XT 17 system.

### Barnes maze

The Barnes maze is a white circular platform with a diameter of 36’’ and elevated 40’’ from the floor. It has 20 holes with a diameter of 2’’ that are equally distributed around the perimeter edge (San Diego Instruments w/ Custom Legs, San Diego, CA). Testing was conducted with a bright light level of ~ 1000 lx on the maze surface. The test lasted for 6 days. On day 0, each mouse was placed on the center of the maze to habituate on the maze and explore the maze freely. An escape box was placed under one of the holes that was not to be used during training and probe testing trials. All other holes were blocked. After 1 min, mouse was gently directed into the escape box. Day 1–4 were for training trials to test the mice’s ability in learning. There were 4 visual cues present on the walls and the escape hole location was rotated between mice. Each mouse was placed on the center of the maze and explore the maze to find and enter the escape box. The trial ended when the mouse entered the escape box or 3 min have elapsed. Training was conducted 4 times per day for 4 consecutive days. Mice were rested 10–15 min between trials. On day 5, mice were given a 1.5 min probe testing trial to test for short-retention memory. All holes on the maze were blocked, including the target hole. Each mouse was placed on the center of the maze to explore and visit the location where the escape box used to be. Three parameters were recorded during training and testing trials, including the primary distance which is the total distance traveled by mice to find the escape hole, the primary latency which is the time that an animal took to encounter the escape hole for the first time, and the search strategies which are defined as random (animals move randomly across the platform until the escape box is found), serial (animals travel through consecutive holes around the periphery of the maze until they find the escape box), and direct (animals navigate directly to the correct quadrant and escape box without crossing the maze center more than once and with less than two errors). The time spent in the target quadrant during probe testing trials was also recorded. Data was analyzed using the EthoVision XT 17 system.

### Immunohistochemistry and immunofluorescence

Mice were anesthetized with intraperitoneal injection of ketamine/ xylazine solution (100 mg/kg/10 mg/kg, Butler Schein Animal Health, Portland, ME), and then perfused intracardially with PBS followed by 4% paraformaldehyde (PFA, 15714, Electron Microscopy Sciences) in PBS (pH 7.4). Cerebella were collected, post-fixed in 4% PFA in PBS for 48 h, and then paraffin embedded in the Comparative Pathology Laboratory at University of Iowa Department of Pathology. Cerebellar were sectioned through the midline vermis at the thickness of 5 µm using a rotary microtome (Leica), mounted onto superfrost/plus slides (48311–703, VWR, West Chester, PA), and processed for immunohistochemistry (IHC) or immunofluorescence (IF) staining as previously described [118]. After deparaffinization, slides were antigen retrieved in 10 mM sodium citrate buffer (pH 6.0) in a high pressure cook for 7 min, blocked with 1% BSA/2% goat serum/0.1% Triton x-100 at room temperature for 1 h, and incubated with primary antibodies at 4 °C overnight. For IHC, slides were treated with 0.3% H_2_O_2_ in methanol for 15 min, then incubated with HRP-conjugated biotinylated secondary antibodies (1:200, Vector Laboratories Inc, Newark, CA) for 1 h and incubated with Avidin–biotin-peroxidase complex (Vector Laboratories Inc). The immunolabeling was visualized using DAB peroxidase substrate kit (SK-4100, Vector Laboratories Inc) and imaged using Olympus IX83 microscope (Olympus, Waltham, MA). For IF, slides were incubated with Alexa fluor-conjugated secondary antibodies (1:200, Life Technologies, Carlsbad, CA) in the dark for 1 h, and counterstained with DAPI (D9542, Sigma-Aldrich, St. Louis, MO). Slides were sealed with VECTASHIELD mounting medium (H-1400, Vector Laboratories Inc) and imaged using the Olympus IX83 microscope (Olympus).

The following antibodies were used for this study: anti-ARMET/ARP (MANF) (ab67271) and anti-ATF6 (ab203119) were from Abcam (Cambridge, MA); anti-Calbindin-D-28 K (C9848) was from Sigma-Aldrich (St. Louis, MO); anti-GRP78 was from Novus Biologicals (Littleton, CO); anti-phosphorylated eIF2α (CST3398), anti-phosphorylated PERK (CST3179), anti-Calbindin (CST13176), and anti-cleaved Caspase-3 (CST9661) were from Cell Signaling Technology (Danvers, MA); anti-XBP1s (658802) was from BioLegend (San Diego, CA); anti-HYOU1 (PA5-27655) was from Invitrogen. HRP-conjugated anti-rabbit (GENA934) and anti-mouse (GENA931) secondary antibodies were from GE Healthcare Life Sciences (Piscataway, NJ). Alexa-488 conjugated anti-mouse (A21202), Alexa-594 conjugated anti-mouse (A11005) and Alexa-594 conjugated anti-rabbit antibodies (A11012) were from Life Technologies (Carlsbad, CA).

### Stereological cell counting

Every twentieth section from the serial sagittal sections across the cerebellar vermis was immunolabeled with anti-Calbindin-D-28 K for PCs. The number of PCs in cerebellar lobule II of each section were counted by unbiased stereology that uses systematic random sampling method for counting. The Optical Fractionator probe was performed on an Olympus BX-51 microscope equipped with ASI motorized stage with XYZ encoder. Stereo Investigator software (v11, MBF Bioscience, Williston, VT) was used to estimate the total number of PCs in lobule II of each brain sample. The contours were drawn manually to mark the PC layer. Based on our previous experience, we used a sampling grid of 800 × 800 μm and a counting frame of 200 × 200 μm for all the brain samples measured, which generated the coefficient of error of 0.07 approximately. We counted the number of PCs at a final magnification of 100x, using the unbiased counting rule that uses two exclusion edges (left and lower) and two inclusion edges (upper and right) on the images while counting.

### Immunoblotting

Protein was extracted from mice brains as described previously [110]. Protein concentration was determined using the DC protein assay according to the manufacture’s instruction (5000111, Bio-Rad Laboratories, Hercules, CA). For immunoblotting, 30 µg protein samples were separated on 12% polyacrylamide gels by electrophoresis and transferred to nitrocellulose membranes and blocked in 5% BSA/1xTBS/0.05% Tween-20 for 1 h at room temperature prior to incubation with primary antibodies at 4 °C overnight. Subsequently, membranes were washed with TBST and incubated with secondary antibodies conjugated to horseradish peroxidase. Blots were developed using the Cytiva Amersham ™ ECL ™ Prime Western Blotting Detection Reagent (Cytiva. RPN2232) on Chemi™Doc imaging system (Bio-Rad Laboratories). Band intensity was quantified using Image Lab software (Bio-Rad Laboratories).

The following antibodies were used for immunoblotting: anti-ARMET/ARP (MANF) (ab67271) was from Abcam (Cambridge, MA); anti-β-Actin (CST3700) was from Cell Signaling Technology (Danvers, MA).

### TUNEL assay

TUNEL assay was performed using the In Situ Cell Death Detection Kit Fluorescein (11684817910, Roche, Indianapolis, IN) according to the manufacturer’s instructions. Slides were deparaffinized and treated with 10 mM sodium citrate buffer (pH 6.0) in a high pressure cook for 7 min. Then, slides were incubated with TUNEL reaction mixture at 37 °C in a humid chamber in the dark for 60 min. Slide incubated in labeling solution without terminal transferase was used as the negative control and slide digested with DNase I and incubated with TUNEL reaction mixture was used as the positive control. The slides were then counterstained with DAPI and imaged with the Olympus IX83 microscope (Olympus).

### Visium spatial transcriptomics analysis (STA)

STA is a next-generation whole transcriptome analysis measuring total mRNA in tissue sections while maintaining the spatial attributes of cells. It is a powerful tool to dissect gene expression heterogeneity in the architecture of intact tissue sections [46]. Tissue processing for Visium spatial transcriptomics (Visium Gene Expression Slide & Reagent Kit, PN-1000184, 10 × Genomics, Pleasanton, CA) was performed in the Iowa Neuroscience Institute (INI) NeuroBank Core following the instruction of the tissue preparation guide demonstrated protocol CG000240. Twelve fresh mouse brains (male control n = 3, male KO n = 3, female control n = 3, female KO n = 3) were dissected and snap frozen in a metal beaker with cooled isopentane in a foam dewar with liquid nitrogen bath. A small piece of the cerebral cortex of each frozen brain was used for RNA extraction. RNA quality was assessed in the Iowa Institute of Human Genetics (IIHG) Genomics division using RNA Integrity Number (RIN) and all samples were confirmed with a RIN above 7. Cerebellum from the 12 samples were cryosectioned through the midline vermis into 10 μm sections. One section of each cerebellum was mounted onto the Visium spatial slides (2000233, 10 × Genomics) within the frames of capture areas. Slides were then processed with methanol fixation, H&E staining and imaging following demonstrated protocol CG000160. Prior to library preparation, the optimal permeabilization condition was determined following the tissue optimization demonstrated protocol CG000238. A total of 18 min of permeabilization was chose based on the optimalization result. After reverse transcription and second strand synthesis, the amplified cDNA samples from the Visium slides were transferred, purified, and quantified for library preparation. Sequencing libraries were prepared by the IIHG Genomics Division, according to the Visium Spatial Gene Expression Reagent Kits User Guide CG000239. The molar concentrations of the indexed libraries were measured using the 2100 Agilent Bioanalyzer (Agilent Technologies, Santa Clara, CA) and combined in a ratio to achieve at least 50,000 sequencing read pairs per tissue covered spot. The concentration of the pool was determined using the Illumina Library Quantification Kit (KAPA Biosystems, Wilmington, MA) and sequenced on the Illumina NovaSeq 6000 genome sequencer using the 28 bp Read 1, 10 bp i7 index, 10 bp i5 index, and 90 bp Read 2 run configuration using SBS v1.5 sequencing chemistry.

Bioinformatic analysis of the Visium data was carried out by the IIHG Bioinformatics division. Raw FASTQ files and H&E images were processed with Space Ranger (10 × Genomics, version 1.3.0) using the mm10-2020-A reference transcriptome. Quality control (QC) showed no quality concerns for all samples. The resulting data matrices were subsequently imported into R (version 4.0.0) and analyzed using the Seurat package (version 4.0.5). Gene expression was normalized and scaled using the SCTransform function. Based on visual inspection of the elbow plot, the first 30 principal components were used in UMAP-based dimensional reduction. The FindClusters function, with a resolution of 0.5, was then used to assign cells to clusters. After confirming that the cell clusters were anatomically accurate by visual inspection, we used the Seurat cluster assignments to create pseudobulk RNA-Seq samples. This aggregation of counts for each individual cluster was done using the aggregateBioVar package (version 0.99.2). These counts were then normalized and transformed using a variance stabilizing transformation (vst) and principal components analysis (PCA) was performed to visualize sample clusters. For statistical analysis of the data, the DESeq2 package (version 1.30.1) was used. In brief, a model incorporating all the experimental factors was created and Wald tests were used to compute statistical metrics. A gene was considered to have a statistically significant change in expression if the false discovery rate (FDR) was less than 10% (adjusted *p* value < 0.1). A gene was considered to have biologically significant change in expression if the base mean was larger than 5 and the |log_2_Fold Change (FC)| was larger than 0.2. Genes found to be differentially expressed under these criteria were used as input for enrichment analysis (iPathwayGuide, Advaita Bioinformatics, Ann Arbor, MI). Visium spots overlay with image were visualized using Loupe browser (10 × Genomics, version 8.1.2). Results from the statistical analysis were visualized using heatmaps (created with pheatmap, version 1.0.12). Venn diagram was generated using the free online tool https://bioinformatics.psb.ugent.be/webtools/Venn/.

### Xenium in situ analysis

Xenium is a probe-based high throughput in situ spatial RNA profiling platform. It allows effective detection for up to 5000 genes on the same tissue section simultaneously [44]. Cerebella from twelve animals (male control n = 3, male KO n = 3, female control n = 3, female KO n = 3) were collected, fixed, and embedded in paraffin as described in the immunohistochemistry and immunofluorescent section. Formalin-fixed paraffin-embedded (FFPE) tissue blocks were sent to the Advanced Genomics Core at the University of Michigan for processing using 10 × Genomics demonstrated protocols CG000578, CG000580, CG000582, and CG000613. Tissue sections (5 µm) were collected onto Xenium slides (3000941, 10 × Genomics) where sections were deparaffinized and decrosslinked. RNA within the tissue was labeled using circularizable DNA probes targeting 300 genes in a standalone custom gene panel (1000648, 10 × Genomics, design ID: HNGXTA). Following ligation, the DNA probes were enzymatically amplified followed by autofluorescence quenching and nuclei staining with DAPI. Xenium slides were loaded into the Xenium Analyzer instrument for imaging and analysis, where fluorescently labeled oligos bound the amplified DNA probes, and samples underwent successive rounds of fluorescent probe hybridization, imaging, and removal to generate optical signatures which were converted into a gene identity. Following the Xenium run, slides were H&E stained, and high-resolution images captured for inclusion in the final data set. Acquired data was transferred off the Xenium instrument and data visualized using the Xenium Explorer (10 × Genomics, version 3.2.0) and third-party analysis tools.

Bioinformatic analysis of the Xenium data was carried out by the Genomics Research and Technology Hub at University of California. Xenium data was downloaded and imported using R package Seurat (version 5.0.3) LoadXenium function. Cell barcodes for regions of interest were obtained from Xenium Explorer and used to subset the raw gene count matrix. Seurat objects for each of the 12 samples were then created and merged ensuring that each gene was expressed in at least 3 cells. After normalization, highly variable genes were then identified using FindVariableFeatures function, and dimension reduction with principal component analysis (PCA) was performed using RunPCA function and uniform manifold approximation and projection using RunUMAP function. To identify differentially expressed genes between different conditions, Wilcoxon rank sum test was used with FindMarkers function. Pseudobulk analysis was performed using DESeq2 (version 1.42.0) where raw counts of cells from the same sample were summed and normalized within DESeq2. Differential gene expression analysis was performed using DESeq2 built in generalized linear models to compare the KO vs control samples. A gene was considered to have a statistically significant change in expression if the false discovery rate (FDR) was less than 10% (adjusted *p* value < 0.1). A gene was considered to have biologically significant change in expression if the base mean was larger than 5 and the |log_2_FC| was larger than 0.2. Volcano and violin plots were generated using GraphPad Prism 10. The enrichment analysis was performed using g:Profiler (version e112_eg59_p19_25aa4782) with g:SCS multiple testing correction method applying significance threshold of 0.05 [53].

### Statistical analysis

All statistical analyses were performed using the GraphPad Prism 10 software unless notified specifically. Data were expressed as mean ± SEM. Differences among experimental groups were analyzed by unpaired t-test, one-way ANOVA, two-way ANOVA, or three-way ANOVA with *p* < 0.05 being considered statistically significant. In cases where significant differences were detected, specific comparisons between treatment groups were examined with the Tukey’s post-hoc test.

## Results

### Characterization of PC-specific MANF KO mouse model

**Fig. 1:**
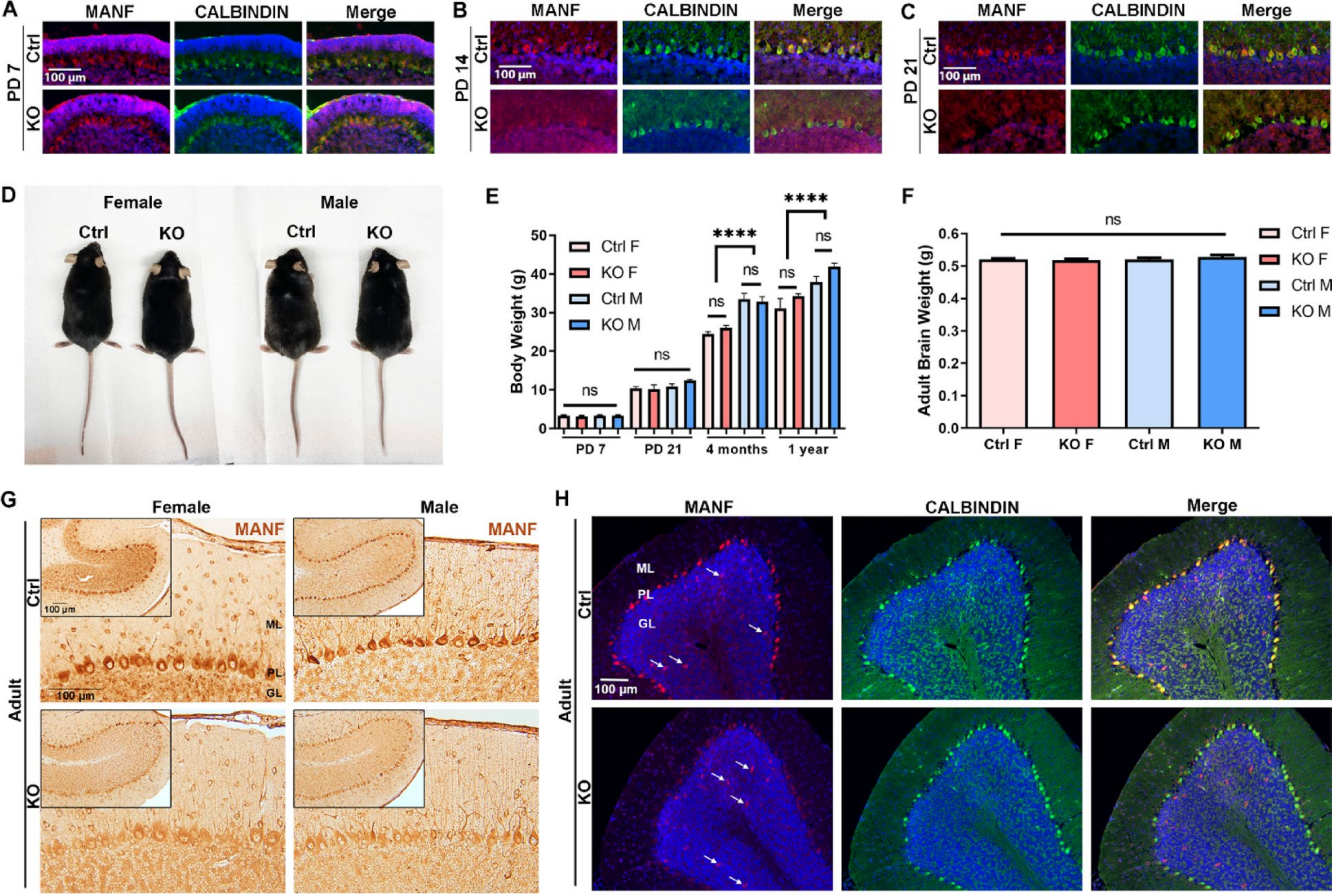
Characterization of PC-specific MANF KO mouse model. **A**–**C**. Representative immunofluorescent images of MANF (red) and PC marker CALBINDIN (green) in the control and KO cerebellum at postnatal day (PD) 7 (**A**), PD 14 (**B**), and PD 21 (**C**). **D**–**F**. Morphology (**D**), body weight (**E**), and brain weight (**F**) of the adult control and MANF KO mice. The data was expressed as mean ± SEM. n = 8–16 per group for body weight; n = 6 per group for brain weight. Two-way ANOVA followed by Tukey’s post hoc test. ****p < 0.0001; ns not significant. **G**. Representative immunohistochemistry images of MANF in the adult control and KO cerebellum. Insets show the entire cerebellum lobule II. **H**. Representative immunofluorescent images of MANF (red) and PC marker CALBINDIN (green) in the adult control and KO cerebellum. Arrows indicated MANF expression in cells located in the granular layer. ML, molecular layer; PL, purkinje cell layer; GL, granule cell layer

MANF is strongly expressed in the developing and adult mouse PCs. Low levels of MANF expression can be detected in the developing PCs at postnatal day (PD) 4. It was increased by PD 7 and expressed strongly in the 4 months old adult cerebellum PCs (Fig. S1A). Immunoblotting of MANF using the 1- and 4-month-old mouse cerebellum lysates demonstrated the expression of MANF in both male and female cerebellum, with female expressing higher levels of MANF than male (Fig. S1B, C). The expression of Cre recombinase in the *Pcp2-Cre* line is driven by the promoter of the mouse *Purkinje cell protein-2* (*Pcp2*) gene that is expressed exclusively in PCs in the brain, thus it is widely used to target gene deletion in PCs [12, 127]. The *Pcp2-Cre* line exhibits onset of Cre recombinase in PCs around PD 6–7. It is fully established by PD 14–15 and remains constant throughout the lifespan of the mice [43, 127]. To confirm MANF knockout in PCs, brains from age matched control (*Manf* ^fl/fl^; *Pcp2-Cre*^−/−^) and KO (*Manf* ^fl/fl^; *Pcp2-Cre*^+/−^) mice at PD 7, 14, and 21 were immunolabeled with anti-MANF antibody and PC marker anti-CALBINDIN antibody. At PD 7, MANF expression was observed in both control and KO PCs, and reduced expression of MANF in KO PCs was not detected yet (Fig. 1A). At PD 14, MANF was significantly reduced in the KO PCs (Fig. 1B). By PD 21, MANF expression was completely absent in the KO PCs (Fig. 1C). We examined the body weight and brain weight of control and KO mice. Regardless of gender and genotype, body weight of control and KO mice were comparable at PD 7 and PD 21 (Fig. 1E). Control and KO male and female mice were raised to adulthood (Fig. 1D). All animals were viable and fertile, with no observable morphological abnormalities. By 4 months and 1 year’s old, male mice were significantly heavier than female mice (Fig. 1E), but no difference in body weight and brain weight was observed between control and KO (Fig. 1D and F). In the adult cerebellum, MANF remained to be absent in the KO PCs in both male and female (Fig. 1G). MANF deficiency was specific to PCs as its expression in the sporadic interneurons in the granular layer was not affected (Fig. 1H, arrows) and its expression in other brain regions such as in the cerebral cortex, hippocampus, and olfactory bulb was not affected neither (Fig. S2).

### MANF deficiency exacerbates binge alcohol exposure induced motor deficits

**Fig. 2:**
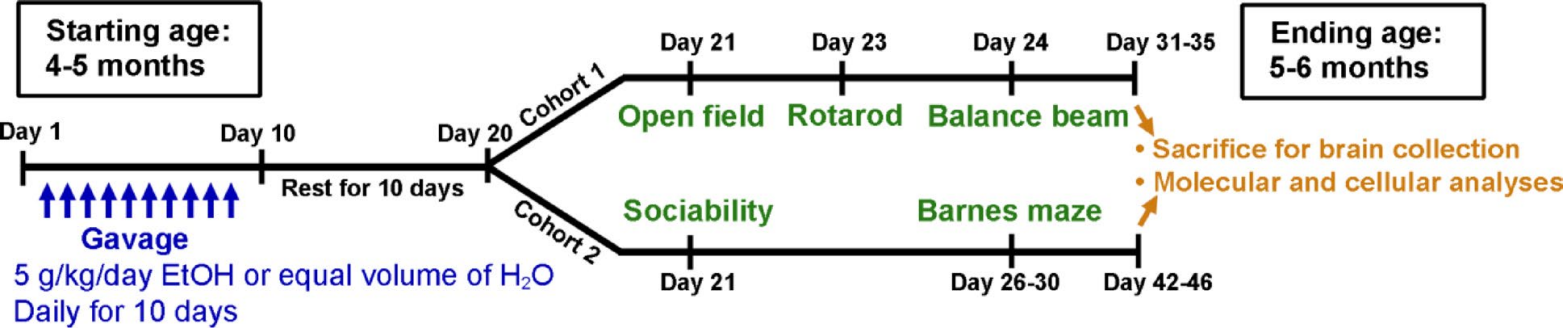
Schematic illustration of the experimental timeline. Both male and female adult mice at the age of 4 to 5 months old received equal volume of H_2_O or ethanol (5 g/kg, 25% ethanol w/v) via intragastric gavage once daily for 10 days. Behavioral tests were performed 10 days after the last ethanol exposure. Cohort 1 animals were tested in open field, rotarod, and balance beam tests. Cohort 2 animals were tested in 3-chamber sociability task and Barnes maze test. After the tests, animals were euthanized, and brains were collected for further cellular and molecular analysis

**Fig. 3:**
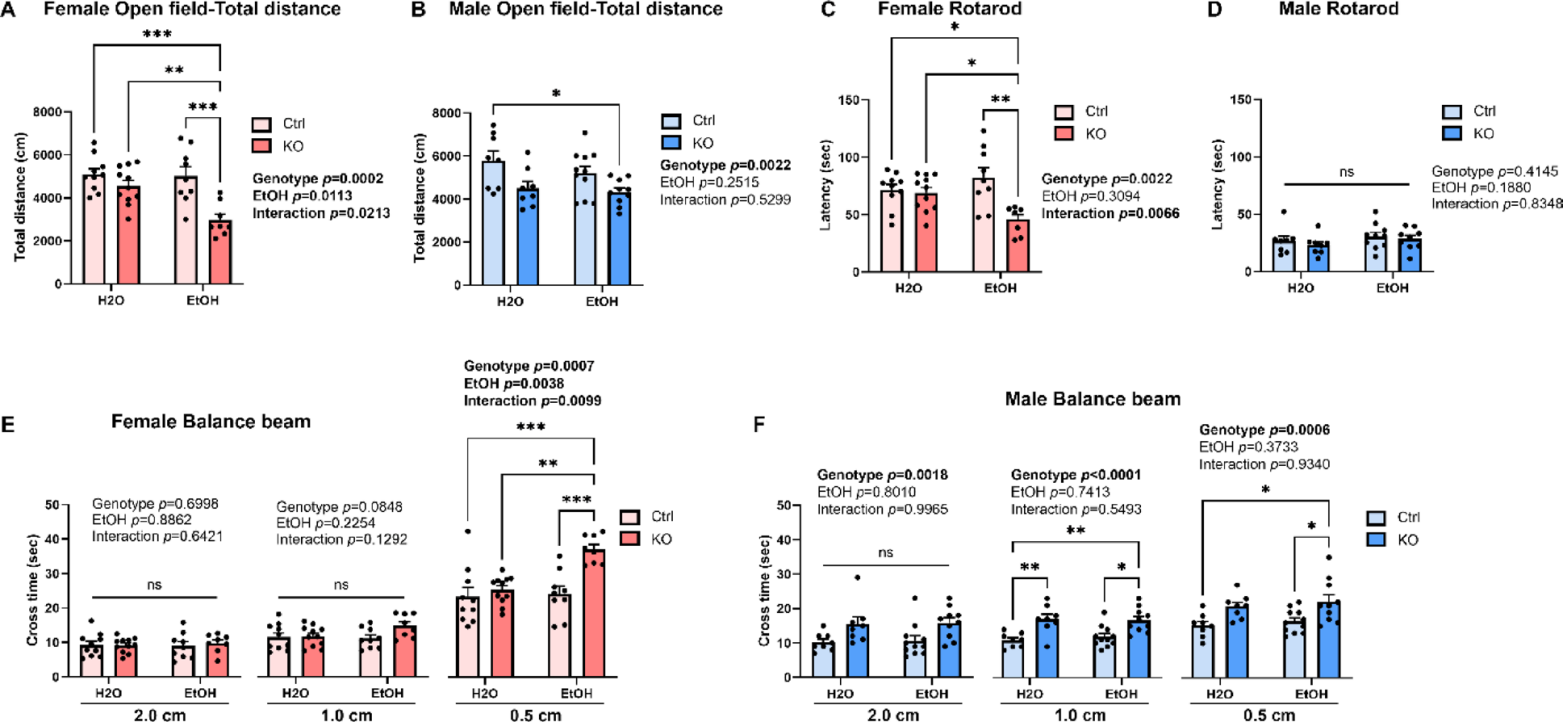
Effects of ethanol exposure on motor functions in PC-specific MANF KO mice. **A**–**B**. Total distance traveled in open field test for water- and ethanol-exposed control and KO female (**A**) and male (**B**) mice. **C**–**D**. The latency to fall from the accelerating rotarod for water- and ethanol-exposed control and KO female (**C**) and male (**D**) mice. **E**–**F**. The time to cross the balance beam for water- and ethanol-exposed control and KO female (**E**) and male (**F**) mice. All data was expressed as mean ± SEM. n = 8–11 per group. Two-way ANOVA followed by Tukey’s post hoc test. Significant main effect *p* values were highlighted in bold; *p < 0.05; **p < 0.01; ***p < 0.001; ns not significant

Control and KO male and female mice at the age of 4–5 months were exposed with either water or ethanol (5 g/kg/day) daily for 10 days through intragastric gavage and their motor function was evaluated in open field, balance beam, and rotarod tests 10 days after the last gavage (Fig. 2). The body weight of all animals was recorded during gavage and behavior tests. Although both male and female control and KO mice showed a significant body weight loss due to ethanol gavage, it recovered to pre-gavage level by the time of behavior tests (Fig. S3). For open filed test, the total distance traveled was recorded. In females, main effects of genotype and ethanol were found with KO females travelled less distance than control females and ethanol-treated animals travelled less than control groups (Fig. 3A). In addition, an effect of genotype and ethanol interaction was observed. Post hoc analysis indicated that ethanol exposed KO female traveled significantly less distance than all the other groups (Fig. 3A). In males, only a main effect of genotype was observed with KO males travelled less distance than control males, while ethanol showed no effects (Fig. 3B). Post hoc analysis showed that ethanol exposed KO males traveled less than water-treated control males (Fig. 3B). Animals were then tested in rotarod test. In females, a main effect of genotype and an interaction of genotype and ethanol treatment were observed. Post hoc analysis indicated a significantly reduced latency to fall from the rotarod for ethanol-treated KO females when compared to all the other groups (Fig. 3C). No difference was observed for males in rotarod test (Fig. 3D). Lastly, animals were tested in balance beam test. In females, although no differences were detected when the beam width was 2.0 and 1.0 cm, there were significant main effects of genotype and ethanol, and an effect of genotype and ethanol interaction when the beam width was as narrow as 0.5 cm (Fig. 3E). Ethanol exposed KO female spent significantly longer time to cross the 0.5 cm beam when compared to other treatment groups (Fig. 3E). For males, there was a main effect of genotype that KO males spent longer time to cross the beam than control males regardless of the beam widths. No main effect of ethanol was observed in male balance beam test, although when the beam width is at 0.5 cm, post hoc analysis found a significant increased crossing time when compared ethanol-treated KO males with control or ethanol-treated control males (Fig. 3F). These results from open field, rotarod, and balance beam tests indicated that PC-specific MANF deficiency impaired motor functions. Binge alcohol exposure exacerbated the motor deficits in female mice with PC-specific MANF deficiency.

### MANF deficiency does not affect binge alcohol exposure induced alteration in social interaction

**Fig. 4:**
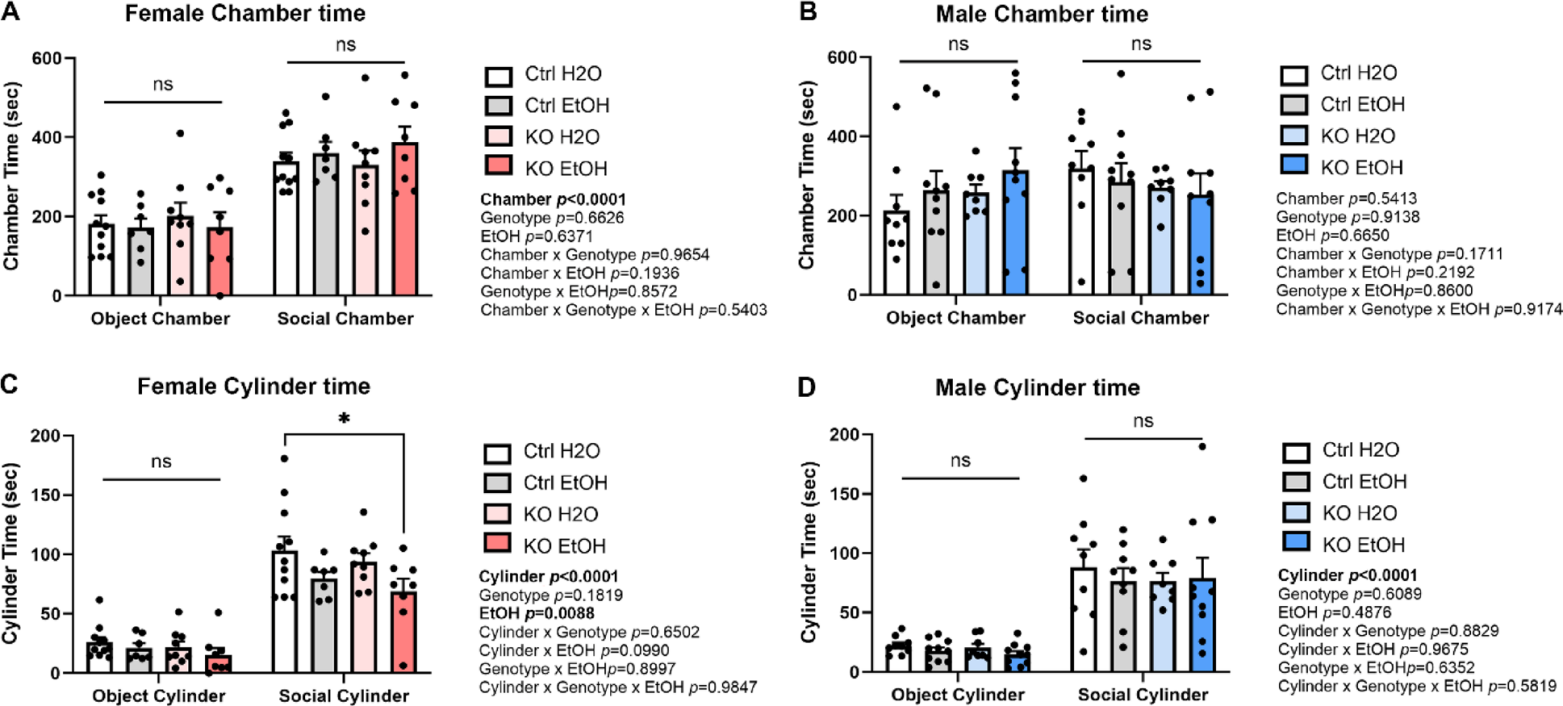
Effects of ethanol exposure on social behaviors in PC-specific MANF KO mice. **A**–**B**. Time spent in the object and social chambers for water- and ethanol-exposed control and KO female (**A**) and male (**B**) mice. **C**–**D**. Time interacting with the object and social cylinders for water- and ethanol-exposed control and KO female (**C**) and male (**D**) mice. All data was expressed as mean ± SEM. n = 8–11 per group. Data was analyzed by three-way ANOVA followed by Tukey’s post hoc test. Significant main effect *p* values were highlighted in bold; *p < 0.05; ns not significant

Binge alcohol drinking is well known to influence sociability in both human and mice [52, 91]. Dysfunction of cerebellum and PCs have also been implicated in social behavior impairments [11, 41, 60]. To test whether social behavior was altered by alcohol and PC-specific MANF deficiency, animals were examined in the 3-chamber sociability test. Female mice stayed significantly longer time in the social chamber than the object chamber (Fig. 4A) and spent significantly longer time interacting with the social cylinder than the object cylinder (Fig. 4C), indicating a preference in social interaction. More importantly, a main effect of ethanol was observed with ethanol-treated females showing reduced social cylinder time than water-treated females (Fig. 4C). For males, although no chamber preference was observed (Fig. 4B), males still exhibit a significant preference of social cylinder over object cylinder (Fig. 4D), indicating that males also prefer social interactions. However, no effect of ethanol nor PCs MANF KO on sociability was observed in male. These results indicated that alcohol impaired social interaction behaviors in the female but not male mice, and it was not affected by PC-specific MANF deficiency.

Animals were also accessed for their anxiety-like behavior and cognitive function in learning and memory, but no effect of alcohol nor MANF KO was observed (Fig. S4–S6).

### Alcohol induces PC degeneration in MANF-deficient females

**Fig. 5:**
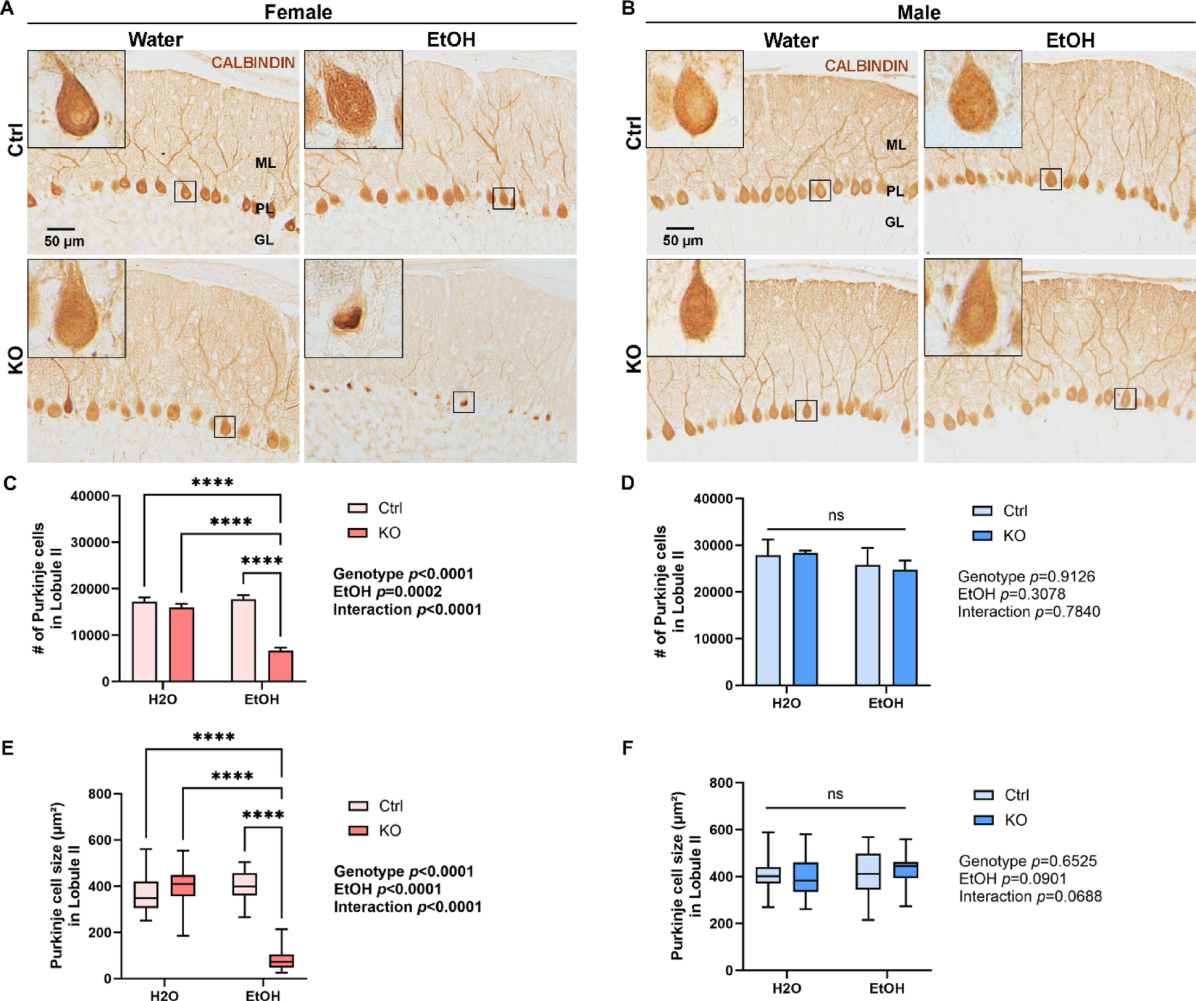
Ethanol induces neurodegeneration in female MANF deficient PCs. **A**–**B**. Representative immunohistochemistry images showing the expression of PC marker CALBINDIN in the adult control and KO cerebellum in female (**A**) and male (**B**) after water- or ethanol-exposure. Insets represent the enlarged view of the PCs framed with the black square. Note the diminished perikaryal staining and predominant intranuclear immunoreactivity of CALBINDIN in the ethanol-treated female KO PCs. **C**–**F**. Quantification of the number (**C**, **D**) and size (**E**, **F**) of PCs in female (**C**, **E**) and male (**D**, **F**) cerebellum lobule II. The data was expressed as mean ± SEM. n = 4 per group. Two-way ANOVA followed by Tukey’s post hoc test. Significant main effect *p* values were highlighted in bold; ****p < 0.0001; ns not significant

After the behavior tests, brains from cohort 1 animals were collected to investigate the cellular and molecular effect of MANF deficiency and alcohol neurotoxicity on PCs. Analyses were performed in the cerebellar vermis lobule II. This region was selected for its consistent anatomical boundaries and well-preserved PC layer across sections. Lobule II also lies within the anterior vermis, an area previously shown to be particularly susceptible to alcohol-induced neurotoxicity [48, 58, 102]. Tissue sections through the cerebellum vermis were immunolabeled with PC-specific marker CALBINDIN to reveal the number and morphology of PCs. Stereological cell counting found a significant reduction in the number of PCs in ethanol-treated KO females (Fig. 5A and C). The remaining PCs in ethanol-treated female KO cerebellum showed a significant decrease in cell body size, indicating PC shrinkage and degeneration (Fig. 5E). Interestingly, we also observed a change in the distribution of CALBINDIN in the ethanol-treated MANF KO PCs. CALBINDIN normally exhibits a diffused expression across the cytoplasm, dendrites, axon, and nucleus in PCs, but in ethanol-treated female KO PCs, there was a diminished perikaryal staining accompanied with predominant intranuclear immunoreactivity (Fig. 5A, insets). In males, however, neither ethanol treatment nor MANF deficiency affected the PC number, PC body size, or CALBINDIN distribution (Fig. 5B, D, and F).

### Alcohol induces ER stress and apoptotic cell death in female MANF-deficient PCs

**Fig. 6:**
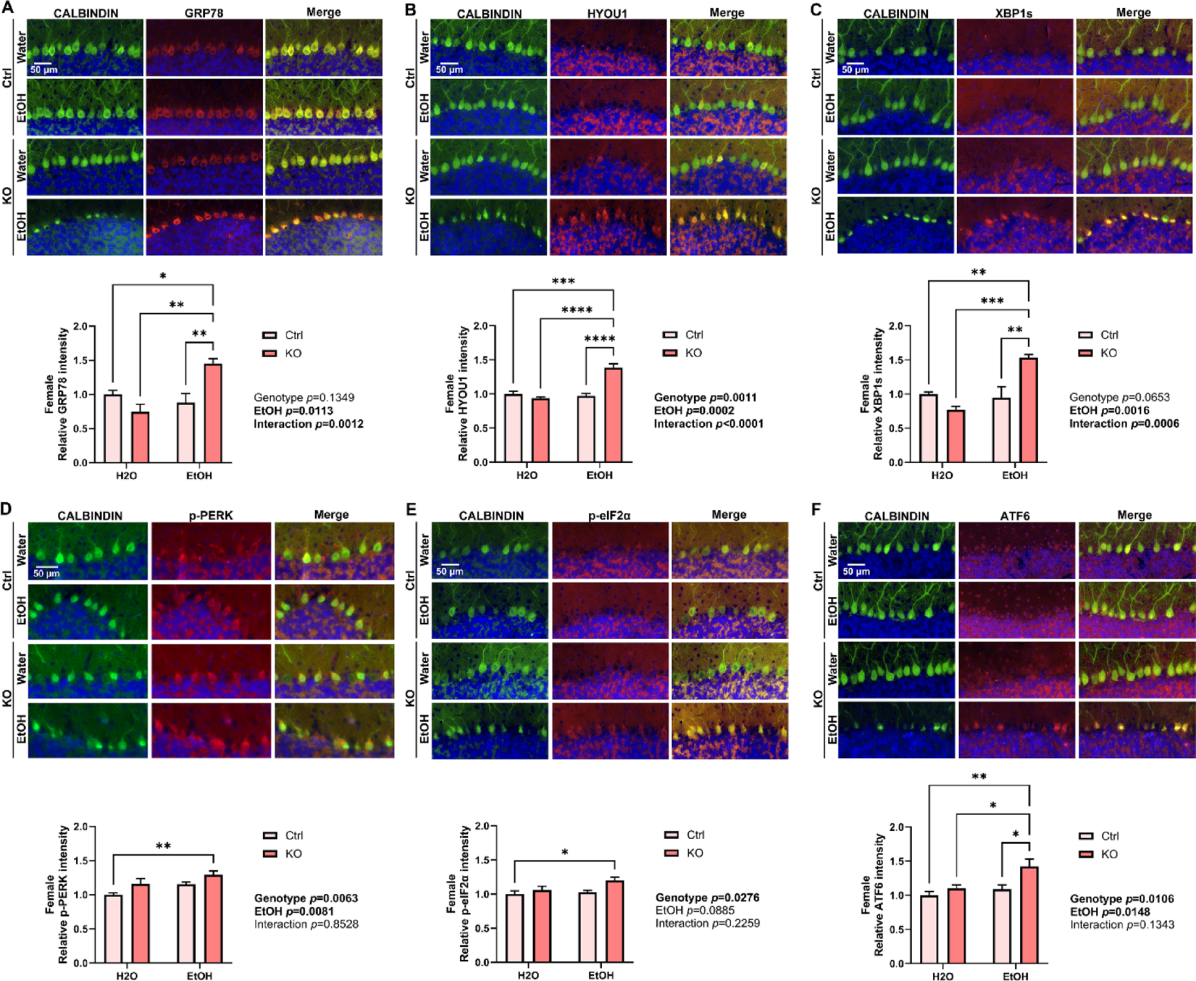
Ethanol induces ER stress in female MANF KO PCs. **A**–**F**. Representative immunofluorescent images and quantifications for the expression of ER stress markers GRP78 (**A**), HYOU1 (**B**), XBP1s (**C**), p-PERK (**D**), p-eIF2α (**E**), and ATF6 (**F**) in water- and ethanol-exposed control and KO female mice cerebellum. PC marker CALBINDIN was co-labeled in green. Quantification of ER stress markers fluorescence intensity was expressed as mean ± SEM. n = 3–4 per group. Two-way ANOVA followed by Tukey’s post hoc test. Significant main effect *p* values were highlighted in bold; *p < 0.05; **p < 0.01; ***p < 0.001; ****p < 0.0001

**Fig. 7:**
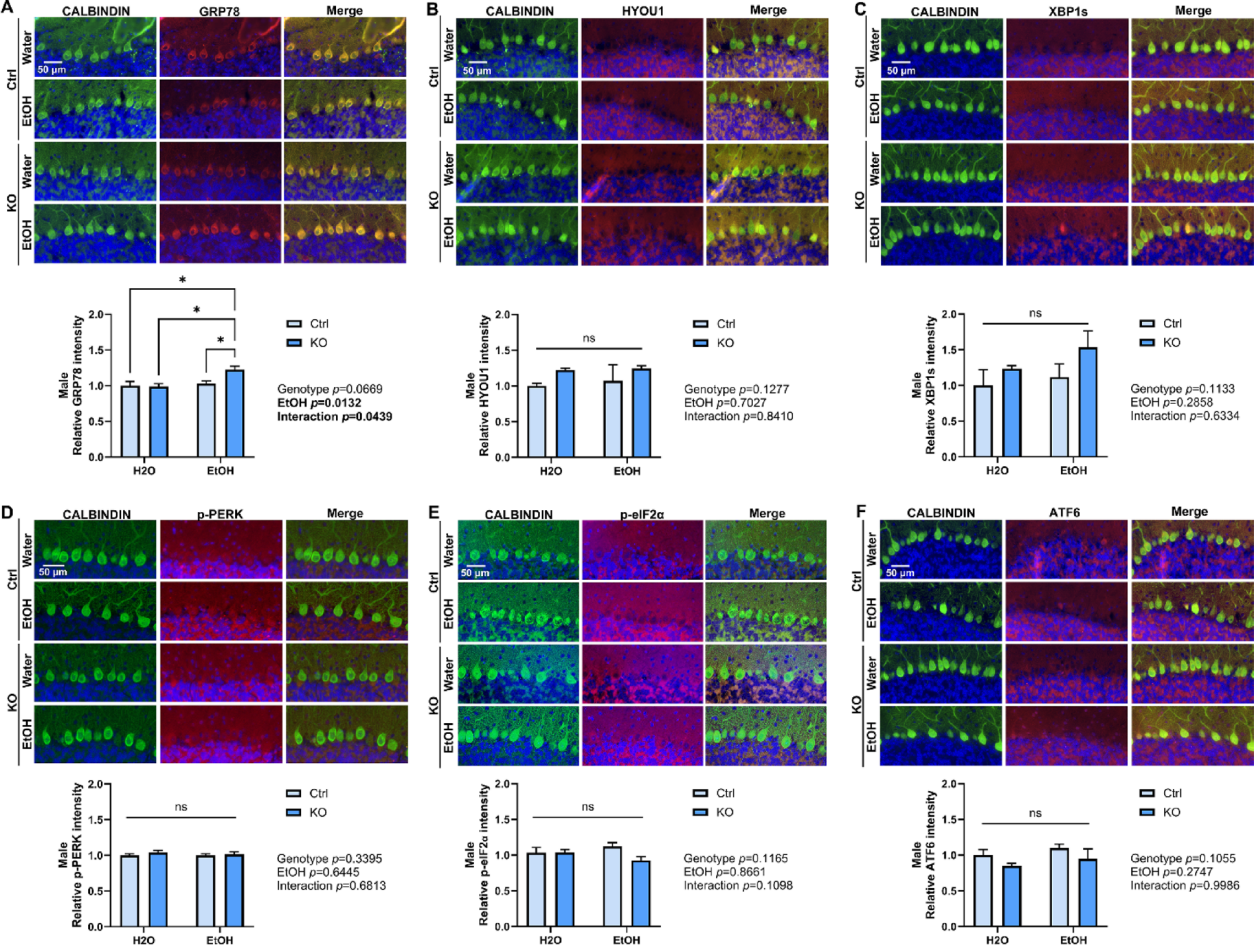
Effects of ethanol exposure on ER stress in male MANF KO PCs. **A**–**F**. Representative immunofluorescent images and quantifications for the expression of ER stress markers GRP78 (**A**), HYOU1 (**B**), XBP1s (**C**), p-PERK (**D**), p-eIF2α (**E**), and ATF6 (**F**) in water- and ethanol-exposed control and KO male mice cerebellum. PC marker CALBINDIN was co-labeled in green. Quantification of ER stress markers fluorescence intensity was expressed as mean ± SEM. n = 3–4 per group. Two-way ANOVA followed by Tukey’s post hoc test. Significant main effect *p* values were highlighted in bold; *p < 0.05; ns not significant

PCs are particularly vulnerable to alcohol-induced disruption in ER homeostasis [21, 22]. We have previously reported that neuronal MANF deficiency exacerbated alcohol-induced ER stress in the brain [113, 114]. To examine if alcohol can cause ER stress in MANF-deficient PCs, tissue sections through the cerebellum vermis were immunolabeled with MANF interacting ER molecular chaperon proteins GRP78 and HYOU1, and UPR proteins XBP1s, p-PERK, p-eIF2α, and ATF6. Results in female showed a significant upregulation of GRP78, HYOU1, XBP1s and ATF6 in ethanol-treated MANF-deficient PCs (Fig. 6A–F). On the contrary, the expression of most markers was not affected by ethanol or MANF deficiency in male PCs, except for GRP78 that it was significantly upregulated in ethanol-treated male MANF-deficient PCs (Fig. 7A–F).

**Fig. 8:**
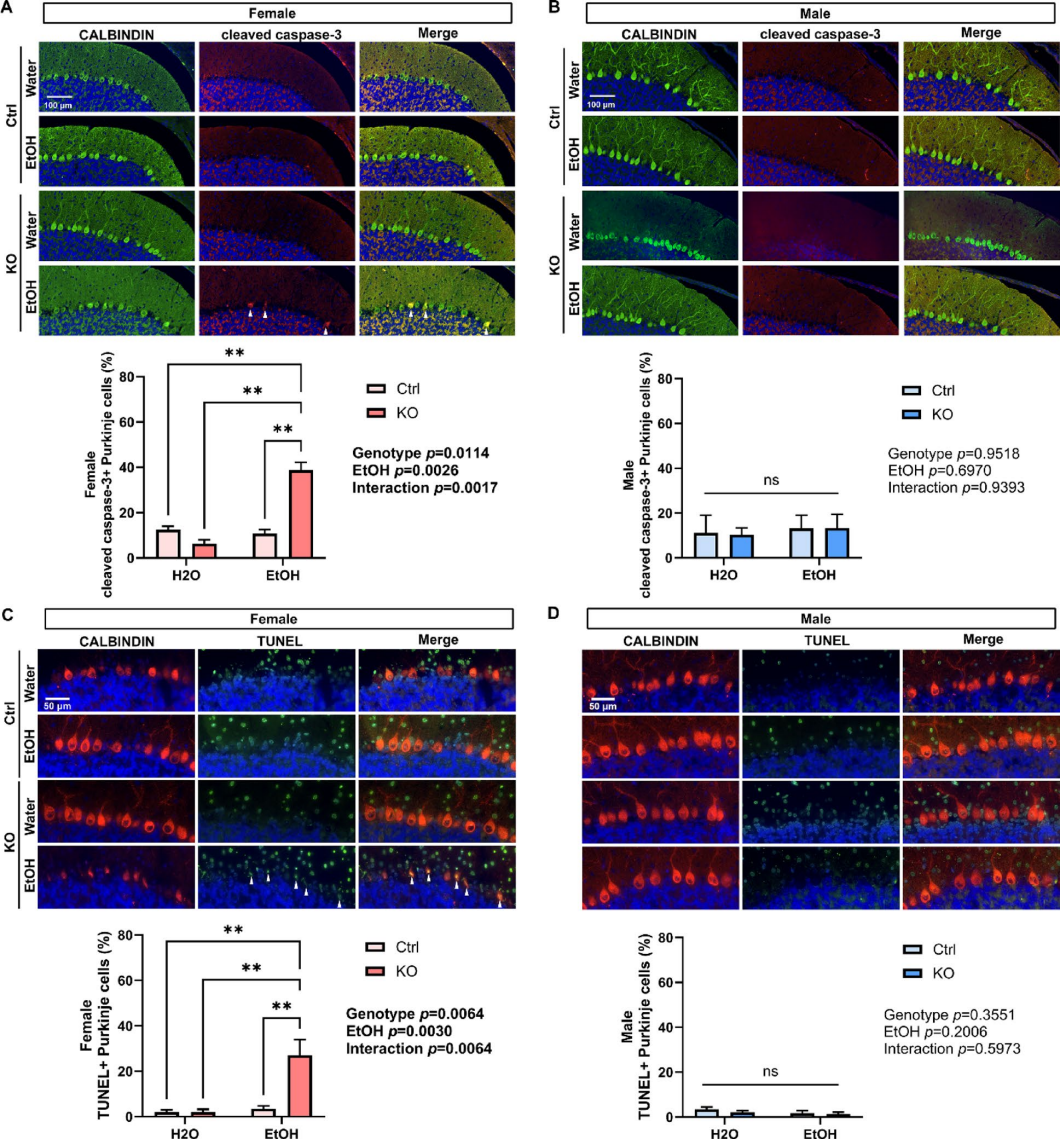
Ethanol induces apoptosis in female but not male MANF KO PCs. **A**–**B**. Representative immunofluorescent images of cleaved caspase-3 (red) and PC marker CALBINDIN (green) in water- and ethanol-exposed control and KO female (**A**) and male (**B**) cerebellum. The number of cleaved caspase-3 positive PCs in lobule II was quantified and expressed as mean ± SEM. n = 3–4 per group. Two-way ANOVA followed by Tukey’s post hoc test. Significant main effect *p* values were highlighted in bold; **p < 0.01; ns not significant. **C**–**D**. Representative images of TUNEL labeling (green) with immunofluorescent co-labeling of CALBINDIN (red) in water- and ethanol-exposed control and KO female (**C**) and male (**D**) cerebellum. The number of TUNEL positive apoptotic PCs in lobule II was quantified and expressed as mean ± SEM. n = 3–4 per group. Two-way ANOVA followed by Tukey’s post hoc test. Significant main effect *p* values were highlighted in bold; **p < 0.01; ns not significant. Arrow heads in A and C indicate cleaved caspase-3 and TUNEL positive PCs, respectively

To further determine whether alcohol and MANF KO result in PC cell death and degeneration, cerebellum vermis sections were immunolabeled with apoptotic marker cleaved caspase-3 and tested in TUNEL assay. In line with alcohol and MANF KO induced female specific PCs shrinkage and UPR activation, we observed a significant increase in the number of cleaved caspase-3 positive and TUNEL positive PCs in ethanol-treated female MANF KO cerebellum (Fig. 8A and C), while no difference in PCs apoptosis was detected in males (Fig. 8B and D).

### MANF deficiency altered the transcriptomic signature in PCs

**Fig. 9:**
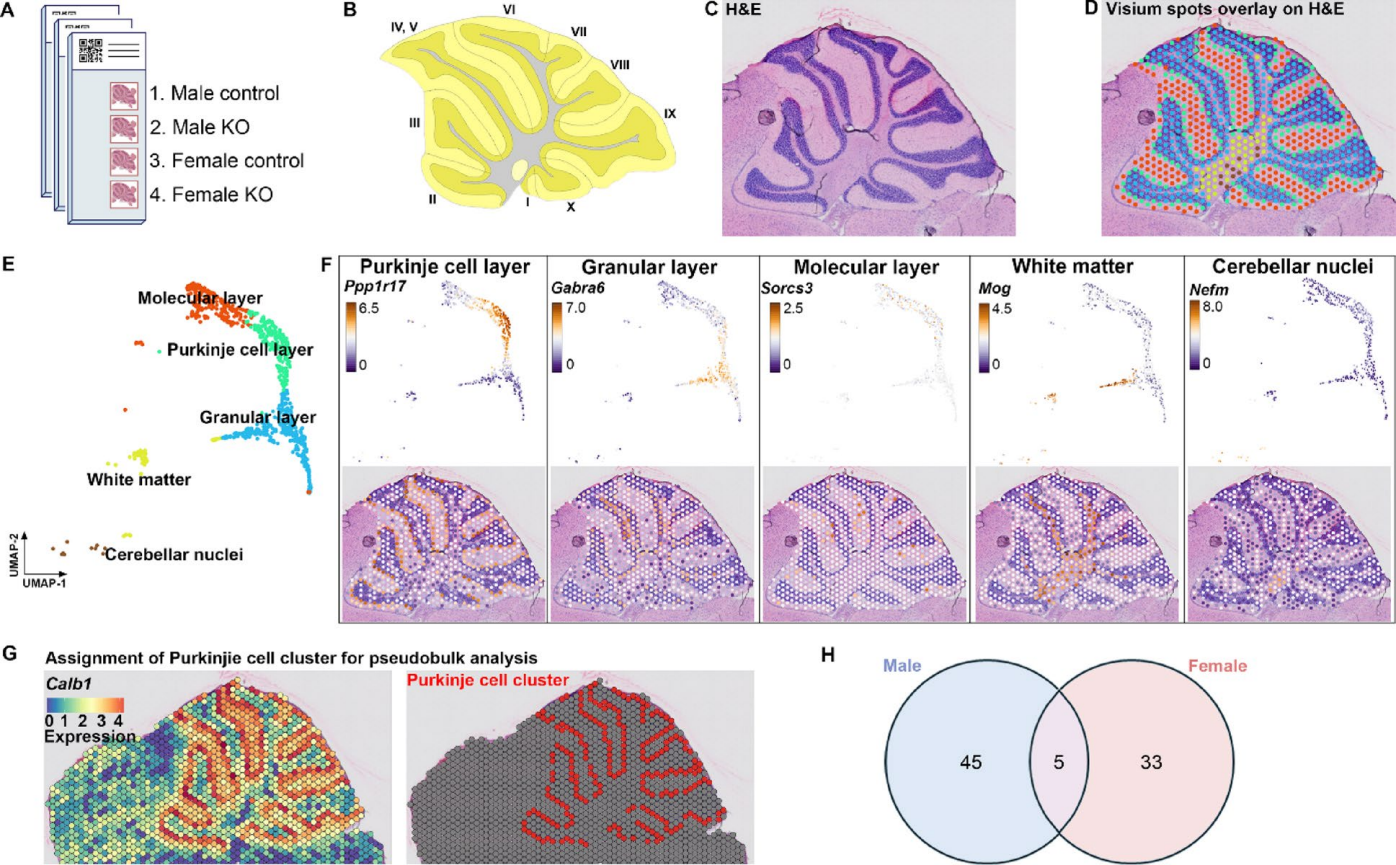
Visium spatial transcriptomics analysis identifies spatially defined tissue clusters in the mouse cerebellum. **A**. Layout of the 3 Visium slides with sagittal sections of the cerebellum vermis from age-matched male and female control and MANF KO adult mice. **B**. Mouse cerebellum vermis sagittal section (adapted screenshot from the Allen Reference Atlas, image 17 of 21, P56, Sagittal. Allen Mouse Brain Atlas, mouse.brain-map.org [1]). Cerebellar lobules were indicated by Roman numerals. **C**. Example of H&E staining image of Visium slide 1, sample 1. **D**. H&E staining image overlayed with unsupervised graph-based clustering of Visium spots in the cerebellum (spots outside of the cerebellum were not shown). **E**. UMAP plot of the Visium spots with annotated tissue clusters. **F**. Expression of selected marker genes for each tissue cluster in the cerebellum. **G**. PC cluster was selected for further pseudobulk analysis based on *Calbindin* (*Calb1*) transcripts distribution in the cerebellum. **H**. Venn diagram showing the male and female overlapping and distinct differentially expressed genes (DEGs) in the PC cluster

**Fig. 10:**
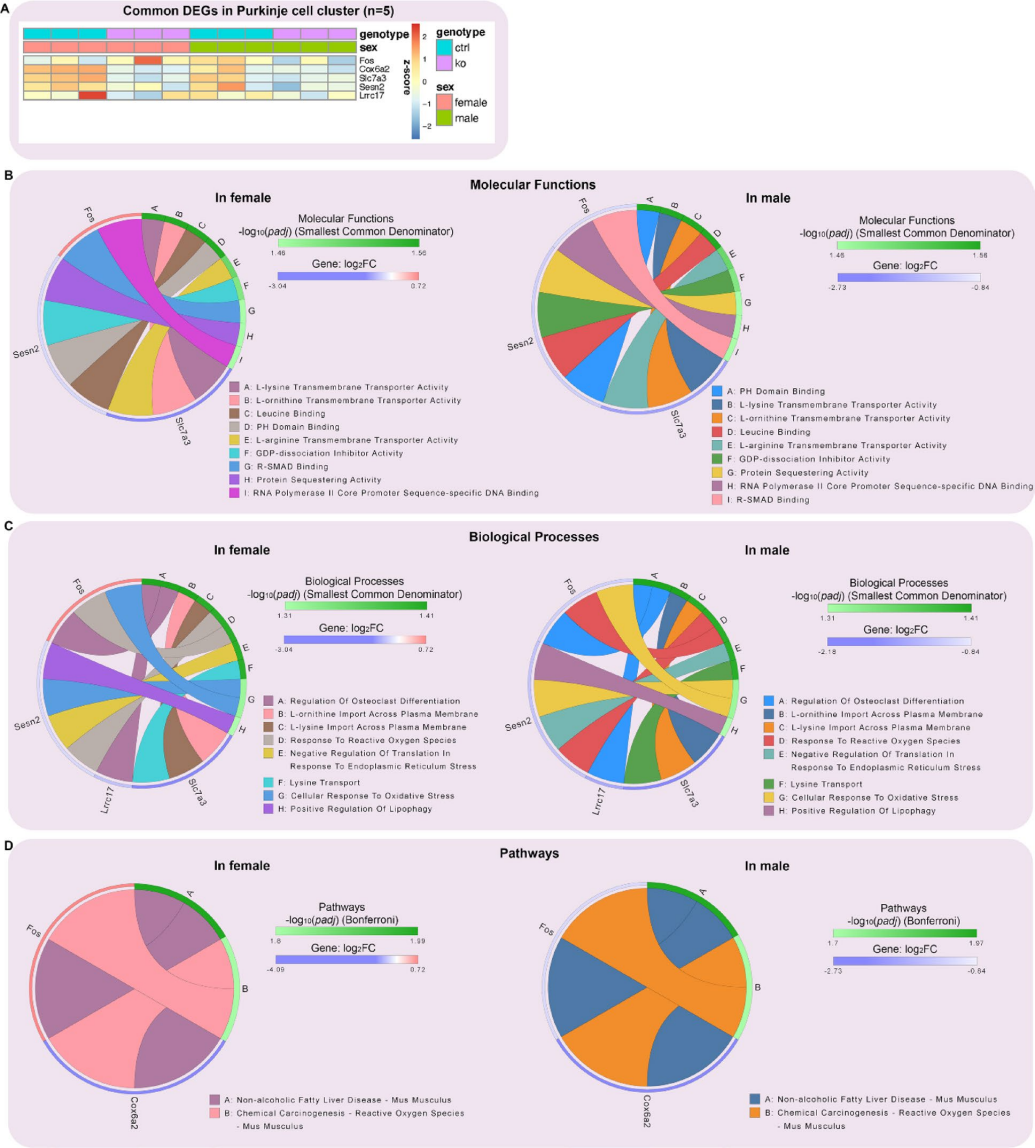
Transcriptional impact of MANF deficiency in both female and male PCs. **A**. Heatmap of differentially expressed genes (DEGs) in control and MANF KO PCs shared in both female and male PC cluster identified by pseudobulk analysis. False discovery rate (FDR)-adjusted *p* value < 0.1, |log_2_ FC|> 0.2, and baseMean value > 5. **B**. Common DEGs enrichment analysis using the Gene Ontology (GO) consortium database for molecular functions. The circular plot shows the top molecular functions and their most important genes in the PCs that were affected by MANF KO. Smallest common denominator-adjusted *p* value < 0.05, that is − log_10_ (*padj*) > 1.3. **C**. Common DEGs enrichment analysis using the GO dataset for biological processes. The circular plot shows the top biological processes and their most important genes in the PCs that were affected by MANF KO. Smallest common denominator-adjusted *p* value < 0.05, that is -log_10_ (*padj*) > 1.3. **D**. Common DEGs enrichment analysis using the Kyoto Encyclopedia of Genes and Genomes (KEGG) database for pathways. The circular plot shows the top pathways and their most important genes in the PCs that were affected by MANF KO. Bonferroni-adjusted *p* value < 0.05, that is − log_10_ (*padj*) > 1.3. Upregulated genes were shown in red, downregulated genes were shown in blue

**Fig. 11:**
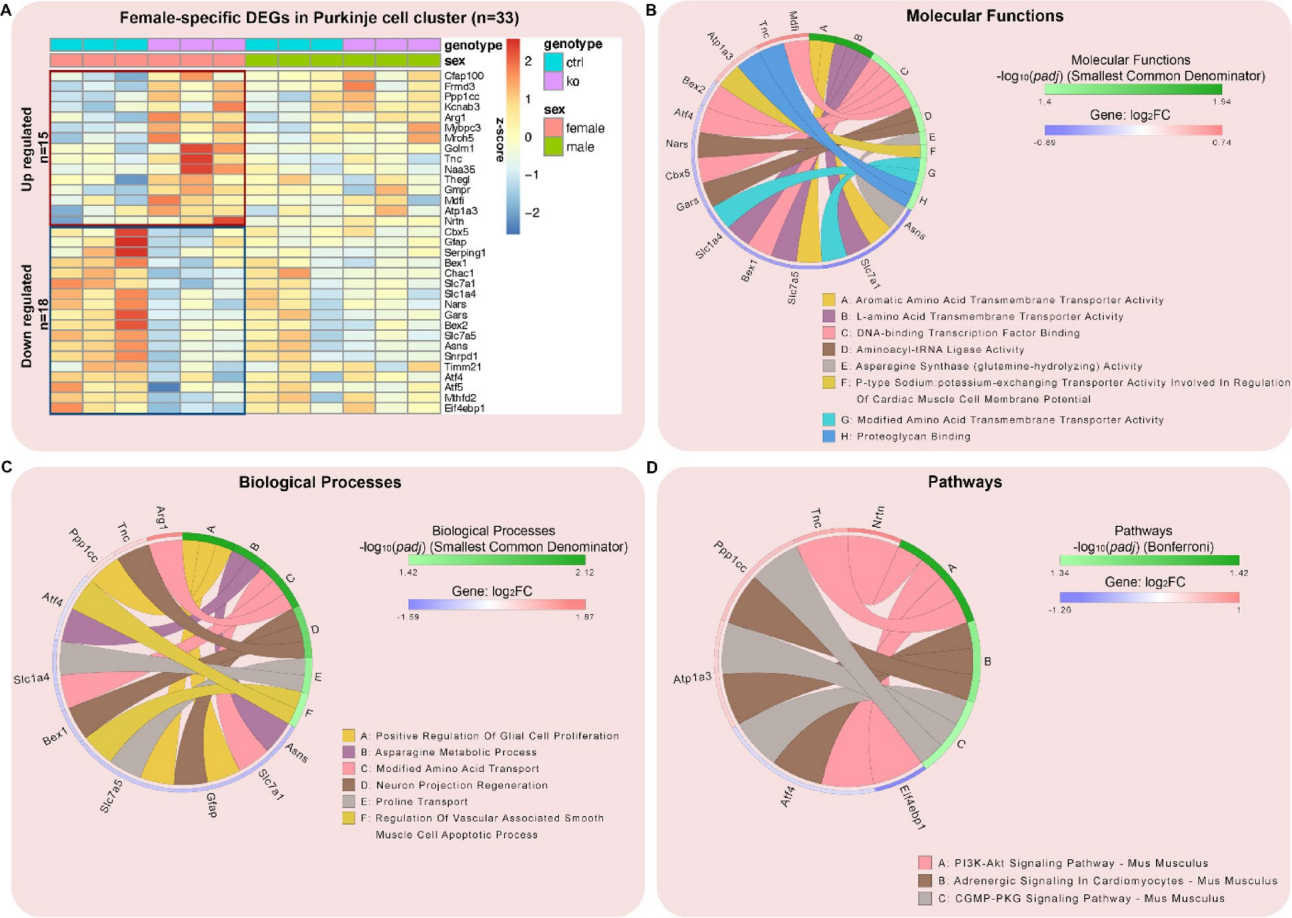
Female-specific transcriptional changes in PCs in response to MANF deficiency. **A**. Heatmap of female specific DEGs in control and MANF KO PCs identified by PC cluster pseudobulk analysis. FDR-adjusted *p* value < 0.1 and |log_2_ FC|> 0.2. **B**. Female-specific DEGs enrichment analysis using the Gene Ontology (GO) consortium database for molecular functions. The circular plot shows the female-specific top molecular functions and their most important genes in the PCs that were affected by MANF KO. Smallest common denominator-adjusted *p* value < 0.05, that is − log_10_ (*padj*) > 1.3. **C**. Female-specific DEGs enrichment analysis using the GO consortium database for biological processes. The circular plot shows the female-specific top biological processes and their most important genes in the PCs that were affected by MANF KO. Smallest common denominator-adjusted *p* value < 0.05, that is -log_10_ (*padj*) > 1.3. **D**. Female-specific DEGs enrichment analysis using the Kyoto Encyclopedia of Genes and Genomes (KEGG) database for pathways. The circular plot shows the female-specific top pathways and their most important genes in the PCs that were affected by MANF KO. Bonferroni-adjusted *p* value < 0.05, that is − log_10_ (*padj*) > 1.3. Upregulated genes were shown in red, downregulated genes were shown in blue

We hypothesized that MANF deficiency may lead to distinct transcriptional alterations in male and female PCs that contribute to the sex-specific susceptibility to alcohol-induced neurotoxicity. To comprehensively understand how MANF deficiency changes the transcriptomic landscape in male and female PCs, we used Visium spatial transcriptomics analysis (STA) to profile gene expression in the cerebellum vermis from age matched 4 months old mice, including 3 control males, 3 KO males, 3 control females, and 3 KO females. Samples were arranged on 3 Visium slides as illustrated in Fig. 9A. PC-specific MANF deficiency was confirmed by MANF immunostaining for all the KO samples before the analysis (Fig. S7A). We detected 1185–3492 Visium spots per sample and 4478–6641 genes per spot, which lead to more than 20,000 genes that were sequenced per sample. Unsupervised clustering identified spots with defined tissue clusters and marker genes expression that recapitulated the cerebellum vermis regions, including the PC layer, granular layer, molecular layer, white matter, and cerebellar nuclei (Fig. 9B–F). Spots for PC cluster were selected based on *Calbindin* (*Calb1*) transcripts distribution in the cerebellum, which were extracted for further pseudobulk analysis (Fig. 9G). PC cluster from female controls were compared with that of female KOs to identify differentially expressed genes (DEGs) in female, and male controls were compared with that of male KOs to identify DEGs in male. We identified 38 DEGs in female and 50 DEGs in male, among which 5 were shared in both female and male, 33 were female specific, and 45 were male specific (Fig. 9H, Table S1). Enrichment analysis indicated that many of the DEGs were involved in protein binding and amino acid transmembrane transportation (Figs. 10–11). Considering that the Visium spots did not provide a single cell resolution, thus the results may be interfered by genes from cells surrounding the PCs such as the Bergmann glia, granular cell, and molecular layer neurons, we further employed Xenium in situ analysis with high resolution single cell information to validate the Visium results and to find new genes that may be potentially overlooked in Visium. A customized 300-gene panel was designed that included most of the DEGs identified in Visium that met the Xenium probe design criteria (Table S2). In addition, it also contained genes encoding MANF interacting proteins reported in previous publications and in the STRING database (version 12.0) [24, 113], genes involved in ER stress/UPR, neuroinflammation, and oxidative stress, and cerebellum cell type marker genes [56] (Table S2). Cerebellum vermis sections from age matched 4 months old mice, including 3 control males, 3 KO males, 3 control females, and 3 KO females, were confirmed with PC-specific MANF KO by immunostaining before proceeding to Xenium (Fig. S7B). We detected 52,901–75,418 cells per sample with 100–132 transcripts per cell. Cell segmentation boundaries were visualized and transcripts of the 300 genes were assigned to each cell in Xenium Explorer (Fig. 13A). Segregated cells were classified into different cell types using unsupervised graph-based clustering and the distribution of cell type marker genes were consistent with the cell clusters (Fig. 13B). Cells in the PC cluster were confirmed with the distribution of the PC-specific gene *Ppp1r17* (protein phosphatase 1 regulatory subunit 17) transcripts expression and were extracted for further pseudobulk analysis (Fig. 13C). We identified 14 DEGs in female PCs and 16 DEGs in male PCs (Fig. 14A, B; Table S3). Transcripts distribution of the top 3 upregulated genes with the most significant *p* values, including *Hspa5*, *Sdf2l1*, and *Cfap100*, were showed in Fig. 14C–K. Among the DEGs, 10 of them were shared by male and female, and all 10 common DEGs were upregulated in KO PCs (Fig. 15A). Enrichment analysis indicated that most of these common DEGs, including *Hspa5*, *Hsp90b1*, *Hyou1*, *P4hb*, *Pdia6*, *Xbp1*, and *Sdf2l1*, were involved in protein folding and response to ER stress (Fig. 15B). *Cfap*100 was the most upregulated gene in the KO PCs in both male and female, however, it was not involved in any of the pathways identified in the enrichment analysis (Fig. 15B). It should be noted that the upregulation of two DEGs, *Xab2* and *Camsap3*, were potentially not due to MANF KO, but because of the cloning of the mouse genome sequence of 3,436,600–3,609,640 on chromosome 8 flanking *Pcp2* gene into the bacterial artificial chromosome while generating the *Pcp2-Cre* line [127]. The intact *Xab2* and *Camsap3* genomic sequence was contained in this genome region, thus potentially resulted in their upregulation in KO. Other than the 10 DEGs shared by female and male, four additional DEGs were found to be female-specific, among which three were ER related, including *Creld2*, *Ddost*, and *Pdia3.* They showed female specific upregulation in MANF KO PCs. *Creld2* had protein disulfide isomerase activity, while *Ddost* and *Pdia3* were in ER protein-containing complex and function in protein processing in ER (Fig. 15C, D). *Kat2b* was downregulated in female KO PCs and was involved in cellular response to nutrient (Fig. 15C, D). Six additional DEGs were male specific. *Camk2n1* and *Derl3* were upregulated in male MANF KO PCs, while *Fos*, *Gabra6*, *Rbfox3* and *Txnip* were downregulated with *Fos* and *Txnip* involved in response to progesterone (Fig. 15E, F). *Camk2n1* encodes CaMKIIN1, an endogenous inhibitor of calcium/calmodulin-dependent protein kinase II (CaMKII). DERL3 protein is a component of the ERAD complex. *Txnip* encodes the thioredoxin-interacting protein which plays a role in cellular stress responses and apoptosis. *Fos* encodes the immediate early transcription factor c-Fos that reflects neural activity. It appears that a subset of male DEGs identified by Xenium may originate from neighboring cell types rather than from PCs. For example, *Gabra6* and *Rbfox3* were identified as upregulated in male MANF KO PCs. *Gabra6* encodes the α6 subunit of the GABA_A_ receptor and *Rbfox3* encodes the RNA-binding Fox 3 protein (also known as NeuN). Both proteins were previously shown to be abundantly expressed in cerebellar granule cells but not in PCs [76, 83]. This finding likely reflects the spatial resolution limits of the assay. When comparing the Visium and Xenium results, the upregulation of *Cfap100* in MANF KO PCs was confirmed in both Visium and Xenium analyses, although it showed up only in female in Visium but in both female and male in Xenium. The downregulation of *Fos* and *Txnip* in male MANF KO PCs were also confirmed in both Visium and Xenium.

**Fig. 12:**
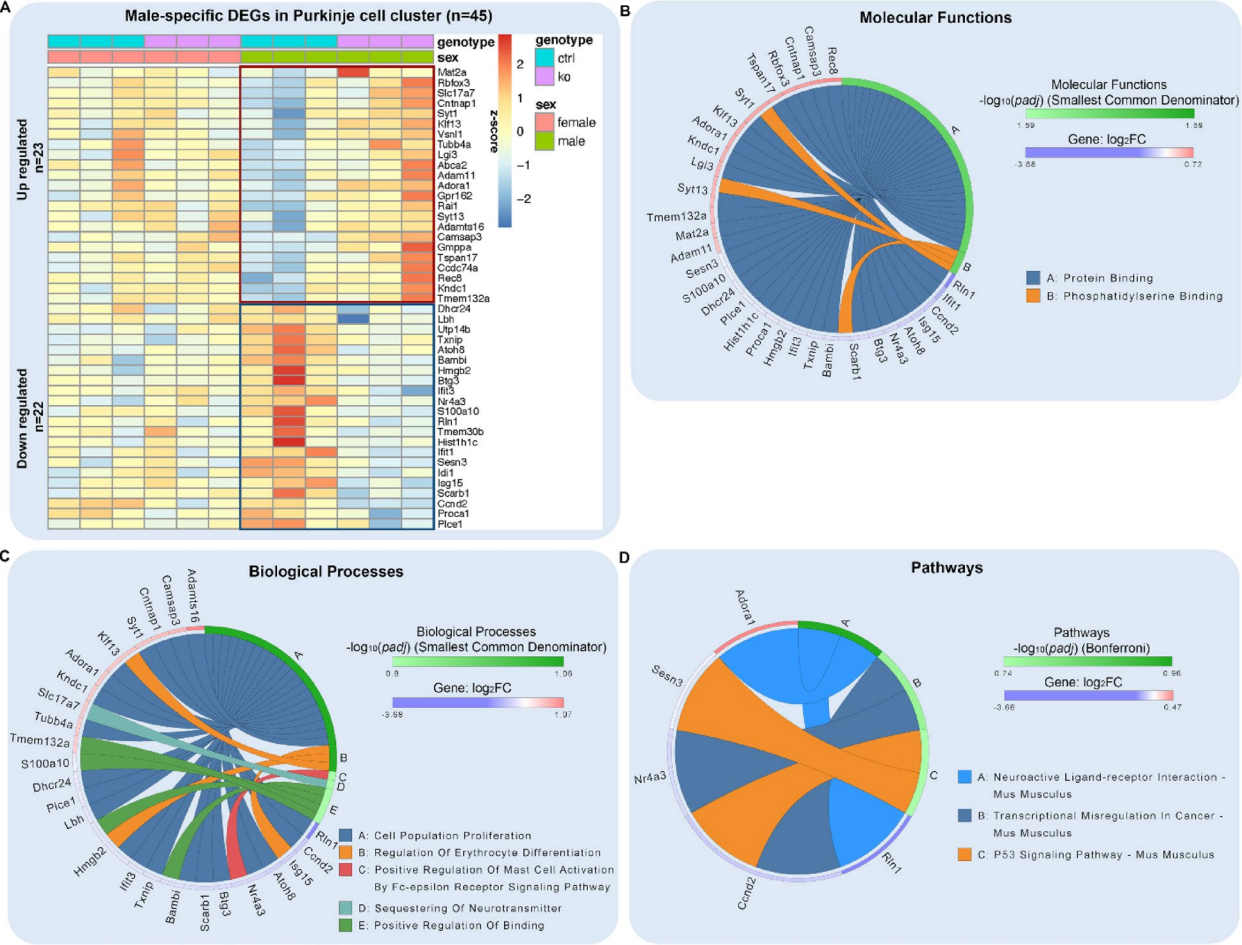
Male-specific transcriptional changes in PCs in response to MANF deficiency.** A**. Heatmap of male specific DEGs in control and MANF KO PCs identified by PC cluster pseudobulk analysis. FDR-adjusted *p* value < 0.1 and |log_2_ FC|> 0.2. **B**. Male-specific DEGs enrichment analysis using the Gene Ontology (GO) consortium database for molecular functions. The circular plot shows the male-specific top molecular functions and their most important genes in the PCs that were affected by MANF KO. Smallest common denominator-adjusted *p* value < 0.05, that is − log_10_ (*padj*) > 1.3. **C**. Male-specific DEGs enrichment analysis using the GO consortium database for biological processes. The circular plot shows the male-specific top biological processes and their most important genes in the PCs that were affected by MANF KO. No processes were identified with the significant smallest common denominator-adjusted *p* values < 0.05. The top 5 processes with the smallest adjusted* p* values were listed. **D**. Male-specific DEGs enrichment analysis using the Kyoto Encyclopedia of Genes and Genomes (KEGG) database for pathways. The circular plot shows the male-specific top pathways and their most important genes in the PCs that were affected by MANF KO. No pathways were identified with the significant Bonferroni-adjusted *p* value < 0.05. The top 3 pathways with the smallest adjusted* p* values were listed. Upregulated genes were shown in red, downregulated genes were shown in blue

**Fig. 13:**
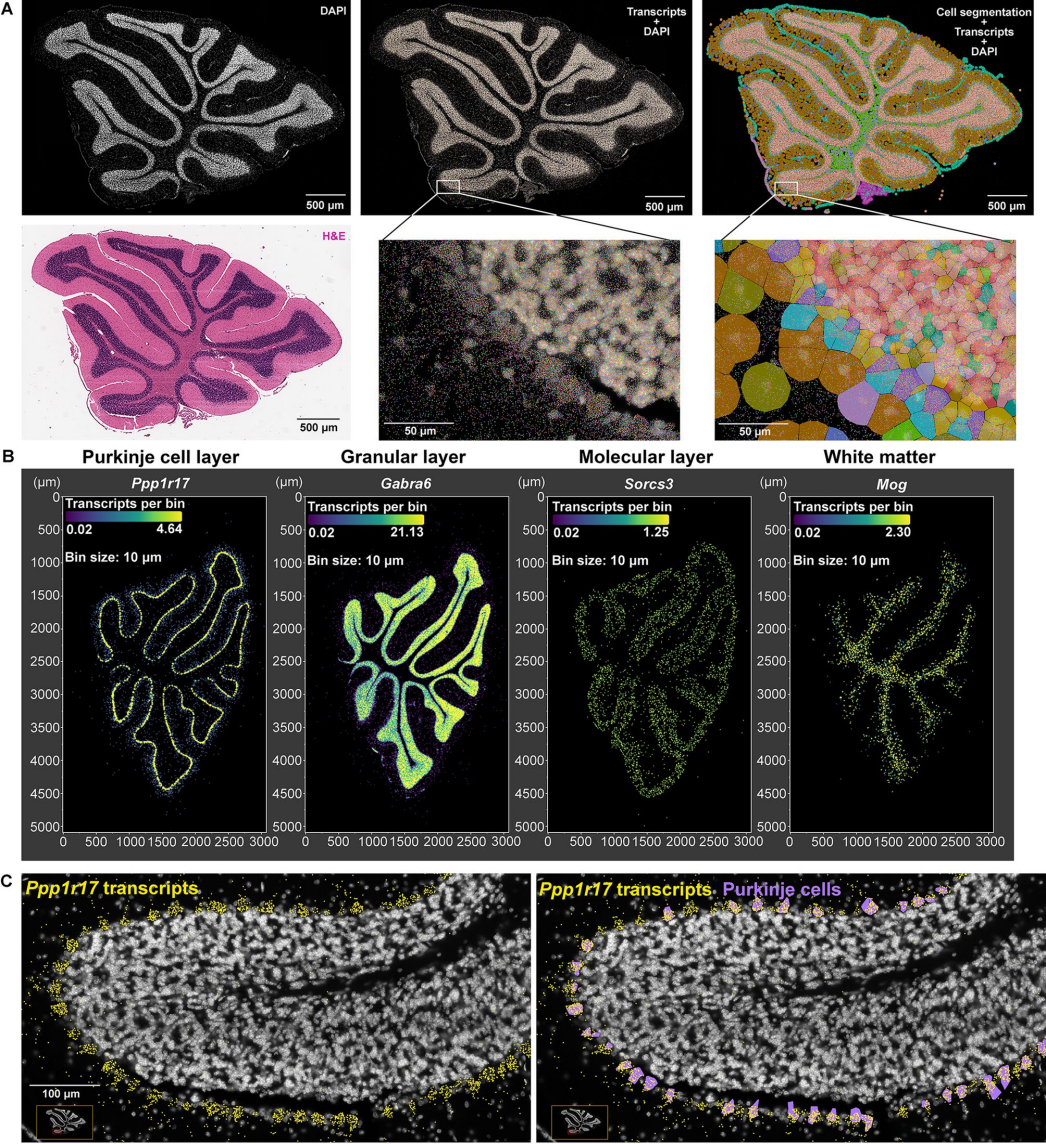
Xenium-based single cell in situ gene expression profile in control and MANF KO cerebellum. **A**. Example of Xenium slide 1, sample 1 cerebellum vermis section stained with DAPI and H&E. Transcripts for 300 genes and unsupervised graph-based clustering of segregated cells were overlayed with DAPI image. Zoom-in images demonstrated individual transcripts as colored dots and cells filled with colors representing different clusters. **B**. Transcript density maps of selected marker genes for distinct cell clusters in the cerebellum. **C**. Cells grouped as the PC cluster (purple) matched the expression of PC-specific gene *Ppp1r17* (yellow dots) in the cerebellum. They were selected for further pseudobulk analysis.

**Fig. 14:**
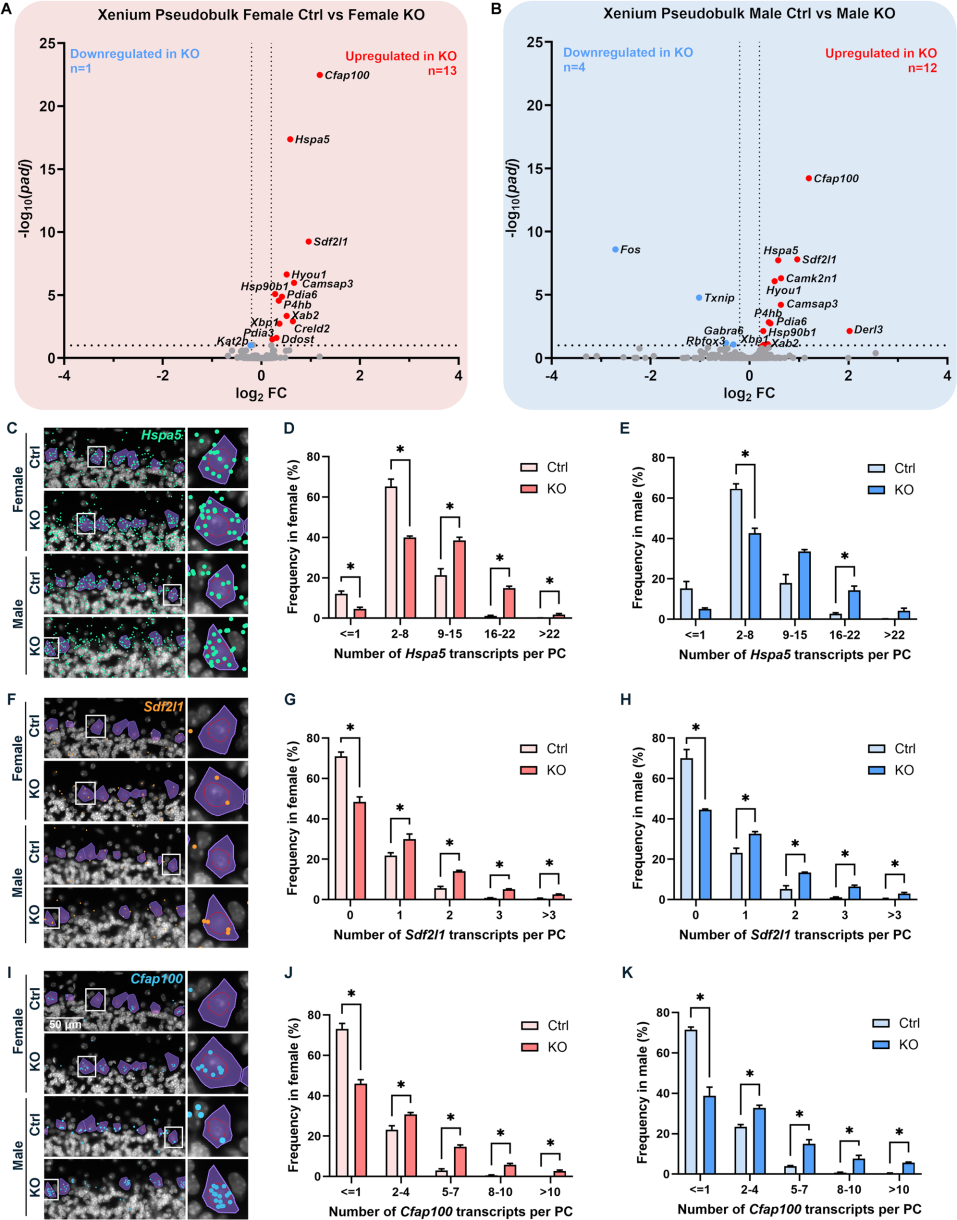
Xenium identifies DEGs in female and male PCs in response to MANF deficiency. **A**–**B**. Vulcano plot showing the -log_10_ (*padj*) and log_2_ FC for the 300 genes in the Xenium gene panel in female (**A**) and male (**B**). FC: fold change. Black dotted lines indicate the threshold of − log_10_ (*padj*) > 1, that equaled to FDR-adjusted *p* value < 0.1, and |log_2_ FC|> 0.2. Red dots: upregulated genes in MANF KO PCs; blue dots: downregulated genes in MANF KO PCs; grey dots: not significantly changed genes. **C**. Representative Xenium results showing the distribution of *Hspa5* (green) transcripts in control and KO female and male PCs (purple). **D**–**E**. Frequency distribution of PCs containing *Hspa5* transcripts in control and KO female (**D**) and male (**E**). **F**. Representative Xenium results showing the distribution of *Sdf2l1* (orange) transcripts in control and KO female and male PCs (purple). **G**–**H**. Frequency distribution of PCs containing *Sdf2l1* transcripts in control and KO female (**G**) and male (**H**). **I**. Representative Xenium results showing the distribution of *Cfap100* (blue) transcripts in control and KO female and male PCs (purple). **J**–**K**. Frequency distribution of PCs containing *Cfap100* transcripts in control and KO female (J) and male (**K**). The data was expressed as mean ± SEM. n = 3 animals per group. Multiple unpaired t tests. *p < 0.05. Red circles within PCs indicated the PC nuclei. PCs framed with the white square were enlarged on the right

**Fig. 15:**
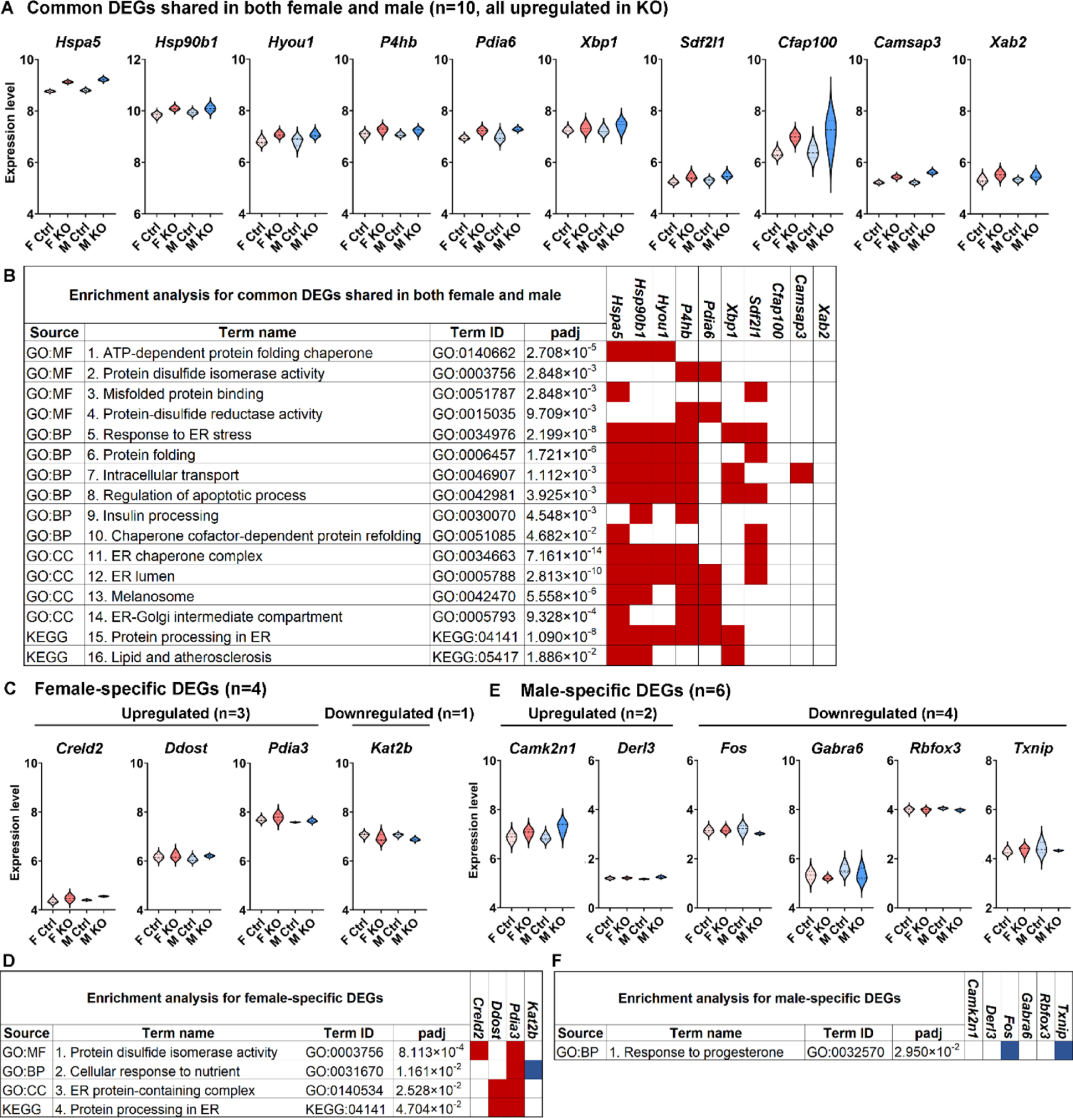
Enrichment analysis of DEGs identified in Xenium. **A**. Violin plots for the expression levels of common DEGs shared in both female and male control and MANF KO PCs. **B**. Enrichment analysis for the common DEGs. **C**. Violin plots for the expression levels of female-specific DEGs in control and MANF KO PCs. **D**. Enrichment analysis for the female-specific DEGs. **E**. Violin plots for the expression levels of male-specific DEGs in control and MANF KO PCs. **F**. Enrichment analysis for the male-specific DEGs. Data sources used in the analysis include Gene Ontology (GO) consortium database and Kyoto Encyclopedia of Genes and Genomes (KEGG) database. MF: molecular function; BP: biological process; CC: cellular component. Adjusted *p* value (padj) was calculated using g:SCS (Set Counts and Sizes) method developed by g:Profiler. Driver GO terms and KEGG terms with padj < 0.05 were listed. The table demonstrated the source of database, term names and IDs with genes involved in each term. Red square: the gene was involved in the term and was upregulated; blue square: the gene was involved in the term and was downregulated

## Discussion

In this study, we generated cerebellar PC-specific MANF KO mouse model and investigated the effect of MANF deficiency on alcohol-induced behavioral impairments and cellular and molecular changes in PCs in the adult mice. We also identified target genes that their expression was altered in response to the loss of MANF, potentially contributing to the phenotype. We found that adult mice with PC-specific MANF deficiency exhibit impaired motor functions. Binge alcohol exposure interacted with MANF deficiency to exacerbate the motor deficits in these animals. Interestingly, female KOs were more sensitive than male KOs to alcohol-induced motor function impairments. In line with the behavior results, alcohol treatment led to UPR activation, alteration in the intracellular distribution of calcium binding protein CALBINDIN, and PC degeneration in female MANF-deficient PCs but not males. Spatial transcriptomics and high throughput in situ analyses demonstrated that MANF deficiency led to sex-specific alterations in the transcriptomic landscape in PCs and triggered the expression of genes involved in protein folding and response to ER stress, potentially contributing to alcohol-induced cellular damages in MANF KO PCs. These results suggests that MANF-deficient PCs are predisposed with a higher risk to UPR activation and ER stress in a sex-dependent manner, contributing to their vulnerability to alcohol neurotoxicity. Our future research will directly determine the role of identified MANF-regulated genes in sex-specific vulnerability to alcohol by manipulating their expression in PCs. We acknowledge that the behavioral and molecular assessments were performed more than 30 days after the final alcohol exposure. It is therefore possible that partial recovery of certain parameters occurred during this interval, which could contribute to the observed sex differences. Future studies incorporating multiple post-exposure time points and blood alcohol clearance measurements will be necessary to determine the temporal dynamics of injury and recovery in male and female mice.

This study highlighted the importance of protein homeostasis in PCs survival and alcohol neurotoxicity. PCs have a high demand for protein synthesis and are vulnerable to disruptions in ER function. Conditional knockout of the major ER chaperone GRP78 in PCs resulted in UPR activation and PC degeneration [111]. Imbalance in protein homeostasis is associated with PC degeneration in various disease models. For example, upregulation of UPR was observed in the *woozy* mutant mouse that exhibit cerebellar ataxia and PC loss due to mutation in the GRP78 nucleotide exchange factor gene *Sil1*, which was associated with the Marinesco-Sjögren syndrome in human [6], demonstrating that perturbation of ER chaperone function in PCs cause ER stress and subsequent PC degeneration [128, 129]. Similarly, in the Purkinje cell degeneration (pcd) transgenic mouse model that exhibit progressive PC degeneration and ataxia, a significant increase in the expression of GRP78 was observed, along with the upregulation of ER stress-induced apoptosis markers CHOP and caspase 12, suggesting that the activation of UPR contributed to PC degeneration in pcd mouse model [57]. ER stress is also associated with PC degeneration in several mouse models of SCA, including SCA type 6, 17, and 22 [37, 40, 107]. In addition, ER-associated degradation (ERAD) is a conserved quality control pathway responsible for the removal of misfolded proteins in the ER. PC-specific deletion of a key ERAD adaptor protein Sel1L (suppressor of lin-12-like 1) led to progressive PC loss in mice [95]. Previously, we have shown that alcohol caused widespread ER stress and UPR activation in the developing and adult mouse brain [50, 112]. Chronic alcohol exposure has been reported to increase the expression of ER stress markers and induce dilation of the smooth ER in PCs in adult and aging rat brain [21–23]. Given the sensitivity of PCs to ER stress, alcohol-induced ER dysfunction and imbalance in protein homeostasis may contribute to the vulnerability of PCs to the toxic effect of alcohol. In this study, binge alcohol exposure or MANF deficiency separately did not induce PC degeneration and UPR activation at the time of assessment (approximately 30–40 days after the last alcohol exposure). However, when combined alcohol with MANF deficiency, significant PC degeneration was observed, especially in females. This may be largely due to the function of MANF in modulating ER stress.

MANF expression and secretion can be induced by ER stressors, indicating that MANF is involved in ER stress related signaling pathways. Various in vitro and in vivo studies have demonstrated that under ER stressed conditions such as cerebral ischemia, hypoxia, and some neurodegenerative diseases, MANF deficiency led to elevated UPR and cell death, while MANF overexpression promoted neuronal survival [7, 28, 79, 103, 123]. Previously using a neuron-specific MANF KO mouse model, we found that MANF KO mice were compromised in ER homeostasis modulation and were more susceptible to alcohol-induced neuronal ER stress and neurodegeneration in the developing and mature mouse brain [113–115]. The current study supported these finding and further demonstrated that MANF deficiency and alcohol exposure synergistically induced ER stress and impaired PCs function.

One interesting phenotype we observed was the intranuclear translocation of CALBINDIN in alcohol-treated female MANF KO PCs. CALBINDIN is a calcium binding protein acts primarily as a cellular Ca^2+^ buffer in PCs. CALBINDIN can regulate neuronal calcium homeostasis and modify calcium-dependent intracellular signaling by buffering calcium and modulating calcium channel activity [2]. CALBINDIN protein normally distributes diffusely in the cytoplasm, dendrites, axon, and nucleus of PCs. Intranuclear CALBINDIN localization is abnormal and has been reported in animal models with hypoxia and chronic morphine treatment [30, 49]. It often reflects excessive nuclear Ca^2+^ influx or nuclear envelope permeability changes, that may regulate gene transcription and activate intranuclear Ca^2+^ signaling in PCs [71]. Nuclear accumulation of CALBINDIN in alcohol-treated female MANF KO PCs may represent a compensatory attempt to buffer excessive nuclear Ca^2+^ levels and protect Ca^2+^-sensitive nuclear processes. The ER is the primary intracellular Ca^2+^ storage organelle in eukaryotic cells. The ER surrounding the nucleus is contiguous with the outer nuclear membrane of the nuclear envelope, referred to as the nucleoplasmic reticulum (NR). Ca^2+^ can enter the nucleus from the cytoplasm through nuclear pores or can be released from the NR via specific channels, such as the ligand-gated Ca^2+^ release channels inositol 1,4,5-trisphosphate receptor (InsP3R) and ryanodine receptors (RyRs) [85, 105]. The dynamic structure of the NR can be altered in pathological conditions [106]. Disruption of Ca^2+^ homeostasis and increased intracellular Ca^2+^ is a key mechanism underlying alcohol-induced neuronal death. Long term alcohol exposure was reported to induce InsP3R upregulation in mouse cerebral cortical neurons [74]. Study using primary cultures of cerebellar granule neurons has demonstrated that alcohol can induce increased intracellular Ca^2+^ and pretreatment with InsP3R inhibitor blocked both alcohol-induced rise in Ca^2+^ and neuronal death [54]. Alcohol-induced CALBINDIN intranuclear translocation in female MANF KO PCs may indicate a dysregulation of Ca^2+^ homeostasis and reflect an adaptive response to excessive nuclear Ca^2+^ accumulation in the female PCs in response to MANF deficiency and alcohol treatment. It is well-established that ER stress destabilizes ER Ca^2+^ homeostasis, leading to Ca^2+^ efflux from the ER [64, 70, 90]. MANF secretion is known to be regulated by intracellular Ca^2+^ level [35]. Alcohol-induced increase in intranuclear CALBINDIN expression in female MANF KO PCs suggests that MANF may be involved in the cellular response to alcohol-induced Ca^2+^ dysregulation. MANF deficiency may potentially alter the intracellular Ca^2+^ signaling in response to alcohol and contribute to alcohol neurotoxicity.

It is interesting that ethanol-induced ER stress, CALBINDIN intranuclear translocation, and neurodegeneration were only prominently observed in MANF-deficient female PCs but not in males. Sex dimorphism in the effect of alcohol have been reported in human and animal studies that females were more vulnerable than males to alcohol-induced organ damages, including the brain [88, 101]. Although women tend to drink less volume and shorter periods of alcohol than men, women exhibit greater volumetric brain loss and increased cognitive impairment than men [25, 39]. The etiology of this sex difference in alcohol neurotoxicity remains poorly understood. Differences in alcohol metabolism, brain structure, hormone, and genetics between male and female may all contribute to the female vulnerability. Research in animal models have demonstrated that for the same amount and period of alcohol exposure, females showed greater neuronal loss [72], severer neuroinflammation [4, 18], different neuroadaptive responses to alcohol withdrawal [38], and distinct transcriptomics alterations [42] than males. We have shown previously using neuronal MANF-deficient adult mice that female, but not male MANF KO mice exhibited an increased locomotor activity and female MANF KO animals were more susceptible to alcohol-induced body weight loss [114]. We also found that alcohol altered the neuronal expression of several UPR and neuroinflammation markers in MANF KO mice in a sex-specific manner [114]. The present findings provide additional evidence to support the sex-specific effect of MANF deficiency on alcohol neurotoxicity, emphasizing the importance to consider sex difference in alcohol research. The level of neurotrophic factors varies in male and female brains. MANF expression in the cerebellum is higher in females than in males, as shown in our supplementary Fig. 1. Other neurotrophic factors such as brain-derived neurotrophic factor (BDNF) is also differentially expressed in the female and male brains [15]. Sex-specific differences in the expression of neurotrophic factors may contribute to the observed female-specific vulnerability of PCs in our model. It is known that BDNF, glial cell line-derived neurotrophic factor (GDNF), and neurotrophin-3 (NT-3) are protective for PCs. The MANF paralog cerebral dopamine neurotrophic factor (CDNF) is also highly expressed in the PCs [66]. Further investigations are needed to determine whether these neurotrophic factors are sex-differentially expressed in PCs, and whether they play a role in protecting male PCs from alcohol toxicity and MANF deficiency.

Our Visium spatial transcriptomic and Xenium in situ analyses results revealed common molecular changes in response to MANF deficiency. Proteins encoded by some of these genes have been identified previously as MANF interacting partners functioning in protein processing and ER stress, including *Hspa5*, *Hyou1*, *P4hb*, and *Pdia6* [24, 35, 120]*.* They were upregulated in MANF KO PCs in both sexes, as revealed in the Xenium analysis. We also found several more ER stress-related genes that were upregulated in MANF KO PCs, including *Hsp90b1*, *Xbp1*, and *Sdf2l1*. These results suggest that an ER homeostasis associated compensatory transcriptional activity occurred in MANF-deficient PCs, although fully compensation is unlikely as when treated with alcohol, female MANF KO PCs still displayed elevated UPR and neurodegeneration comparing to controls. Xenium result also identified a gene *Cfap100* that was upregulated in both male and female MANF KO PCs, but its function may be irrelevant to ER stress. CFAP100 is cilia and flagella associated protein (CFAP) 100, also known as coiled-coil domain-containing 37 (CCDC37). Little is known about the function of CFAP100 in the brain, although its expression was detected in the cerebellum, medulla, and hippocampus in a genome-wide atlas of gene expression in the adult mouse brain [59]. Here, we are the first to report *Cfap100* expression in the mouse PCs. CFAP100 was believed to bind to microtubule and its upregulation may lead to microtubule disorganization [94]. Disorganization of microtubules and defects in primary cilia are both associated with PC degeneration and impaired PC function [13, 62, 68]. *Cfap100* upregulation in MANF KO PCs suggests a potential effect of MANF deficiency on the disruption of microtubule or primary cilium in PCs. Visium results showed several DEGs that were downregulated in MANF KO PCs in both male and female, including *Cox6a2*, *Slc7a3*, *Sesn2*, and *Lrrc17*. *Cox6a2* encodes a subunit of cytochrome c oxidase (complex IV) that is involved in mitochondrial oxidative phosphorylation. Interestingly, a recent study reported that *Cox6a2* was upregulated in cardiomyocytes of myocardial cell-specific MANF KO mice [108]. This contrasting regulation suggests that MANF may influence mitochondrial gene expression in a cell type-dependent manner.

More importantly, we identified sex-specific transcriptional alterations in response to MANF deficiency, which may underlie the female-specific susceptibility to alcohol in MANF-deficient PCs. MANF-IRE1α interaction attenuated UPR and was required for the pro-survival activity of MANF [55]. TXNIP is a key IRE1α downstream target, mediates oxidative stress and apoptosis under ER stress conditions [17]. Interestingly, our Visium and Xenium results both indicated a downregulation of *Txnip* in male but not female MANF KO PCs. This suggests that the IRE1α-TXNIP axis may be selectively suppressed in males, potentially serving as a compensatory mechanism to limit oxidative and apoptotic stress in the absence of MANF. The lack of such adaptation in females could contribute to their increased vulnerability to ethanol-induced PC damage. In addition, we found a significant downregulation of the immediate early gene *Fos* in male MANF KO PCs in both Visium and Xenium analysis while, intriguing, it was significantly upregulated in female MANF KO PCs in Visium result. *Fos* gene encodes an immediate early transcription factor c-Fos that is rapidly induced by neuronal activity, cellular stress, and nuclear Ca^2+^ signaling [89]. Increased *Fos* expression has been associated with PC degeneration in disease states such as in the pcd mice [34] and in a kainic acid-mediated neural injury mouse model [92]. As discussed above, the observed intranuclear translocation of CALBINDIN indicates that ethanol increases nuclear Ca^2+^ selectively in female MANF KO PCs. *Fos* upregulation in female KO PCs suggests that, synergistically, MANF deficiency also enhances nuclear Ca^2+^ signaling specifically in females. The male-specific downregulation of *Fos* may partially explain the relative resilience of male MANF-deficient PCs to ethanol-induced degeneration compared with females. It is unclear why male MANF KO PCs exhibit reduced *Fos* expression instead. It may be related to another interesting DEG associated with Ca^2+^ homeostasis, *Camk2n1*. *Camk2n1* is upregulated in male but not female MANF KO PCs. *Camk2n1* encodes CaMKIIN1, an endogenous inhibitor of calcium/calmodulin-dependent protein kinase II (CaMKII) [16]. CaMKII is a highly abundant neuronal kinase that transduces Ca^2+^ signals and regulates PC synaptic plasticity and long-term potentiation [100, 130]. Elevated *Camk2n1* expression in male MANF KO PCs likely reflects reduced CaMKII activity. It could be a compensatory mechanism in MANF-deficient male PCs to dampen ER Ca^2+^ release and reduce nuclear Ca^2+^ signaling, hence reduced *Fos* expression in male. This may also partially explain why the increased intranuclear CALBINDIN translocation was apparent only in female MANF KO PCs but not males after alcohol treatment. Together, these results suggest that MANF deficiency disrupts ER Ca^2+^ homeostasis and augments Ca^2+^ transfer to the nucleus, leading to aberrant activation of Ca^2+^-dependent transcriptional activities in a sex-dependent manner, contributing to sex-specific susceptibility to alcohol neurotoxicity.

It is worth noting that the transcriptional changes observed in MANF-deficient PCs may not reflect the same alterations at the protein level. For example, *Hyou1* and *Xbp1* were upregulated in both male and female MANF KO PCs detected by Xenium, but the manifestation at the protein level only became evident in ethanol-treated females in immunofluorescence analysis. This observation suggests that the transcriptional differences identified under baseline conditions may be differentially translated in a sex-dependent manner and it may be further modulated by alcohol exposure. One limitation of our study is that many of the DEGs are not confirmed at protein level and whether they are altered in ethanol-treated PCs is not tested. Future studies will include protein-level validation and functional assays to determine how these transcriptional changes contribute to the female-specific MANF KO PCs vulnerability to alcohol.

## Conclusions

In conclusion, the current findings demonstrate that MANF-deficient PCs are more susceptible to binge alcohol exposure induced ER stress and neurodegeneration in the adult brain in a sex-dependent manner. Such vulnerability is particularly increased in MANF-deficient female PCs. We identified overlapping and sex-specific differentially expressed genes in the adult PCs, indicating an alteration in the PC transcriptomics landscape in response to MANF deficiency. Many of the altered genes are involved in protein binding, amino acid transmembrane transportation, and facilitating protein folding. Some sex-specific genes may contribute to the adaptive regulation of Ca^2+^ homeostasis in response to MANF deficiency. Our study has established a valuable animal model to investigate the role of MANF in PC structure and function as well as molecular mechanisms underlying alcohol-induced PC degeneration. Further studies are to focus on the biological function of the altered genes, and how they contribute to the increased susceptibility of MANF-deficient neurons to alcohol neurotoxicity in the female brain.

## Supplementary Information

Below is the link to the electronic supplementary material.

Supplementary Table 1

Supplementary Table 2

Supplementary Table 3

Original immunoblot for supplementary figure 1 4-month beta actin

Original immunoblot for supplementary figure 1 1-month beta actin

Original immunoblot for supplementary figure 1 1-month MANF

Original immunoblot for supplementary figure 1 4-month MANF

Supplementary Figures

## Data Availability

No datasets were generated or analysed during the current study.

## Abbreviations

ADP: Adenosine diphosphate
ATF6: Activating transcription factor 6
AUD: Alcohol use disorder
BAC: Blood alcohol concentration
BDNF: Brain-derived neurotrophic factor
Calb1: Calbindin
CCDC37: Coiled-coil domain containing 37
CDNF: Cerebral dopamine neurotrophic factor
CFAP: Cilia and flagella associated protein
DAPI: 4ʹ,6-Diamidino-2-phenylindole
DEGs: Differentially expressed genes
ERAD: ER-associated protein degradation
ER: Endoplasmic reticulum
FC: Fold change
FDR: False discovery rate
FFPE: Formalin-fixed paraffin-embedded
GDNF: Glial cell line-derived neurotrophic factor
GRP78: 78-KDa glucose-regulated protein
H&E: Hematoxylin and eosin
HRP: Horseradish peroxidase
HYOU1: Hypoxia upregulated 1
IF: Immunofluorescence
IHC: Immunohistochemistry
InsP3R: Inositol 1,4,5-trisphosphate receptor
IRE1: Inositol-requiring enzyme 1
KO: Knockout
MANF: Mesencephalic astrocyte-derived neurotrophic factor
NR: Nucleoplasmic reticulum
NT-3: Neurotrophin-3
PC: Purkinje cell
PCA: Principal components analysis
Pcd: Purkinje cell degeneration
Pcp2: Purkinje cell protein 2
PCR: Polymerase chain reaction
PD: Postnatal day
PERK: Pancreatic ER kinase-like ER kinase
PFA: Paraformaldehyde
Ppp1r17: Protein phosphatase 1 regulatory subunit 17
QC: Quality control
RIN: RNA integrity number
RyRs: Ryanodine receptors
SER: Smooth endoplasmic reticulum
SCA: Spinocerebellar ataxia
SCG: Superior cervical ganglion
Sel1L: Suppressor of lin-12-like 1
STA: Spatial transcriptomics analysis
UPR: Unfolded protein response
XBP1: X-box binding protein 1
