# Supplementary figures and images for: Spatial transcriptomics analysis uncovers ER stress in MANF-deficient Purkinje cells underlying alcohol-induced cerebellar neurodegeneration in mice
Source: Acta Neuropathol Commun. 2025 Dec 3;14:10. doi: 10.1186/s40478-025-02162-1 (PMC12781712; doi:10.1186/s40478-025-02162-1)

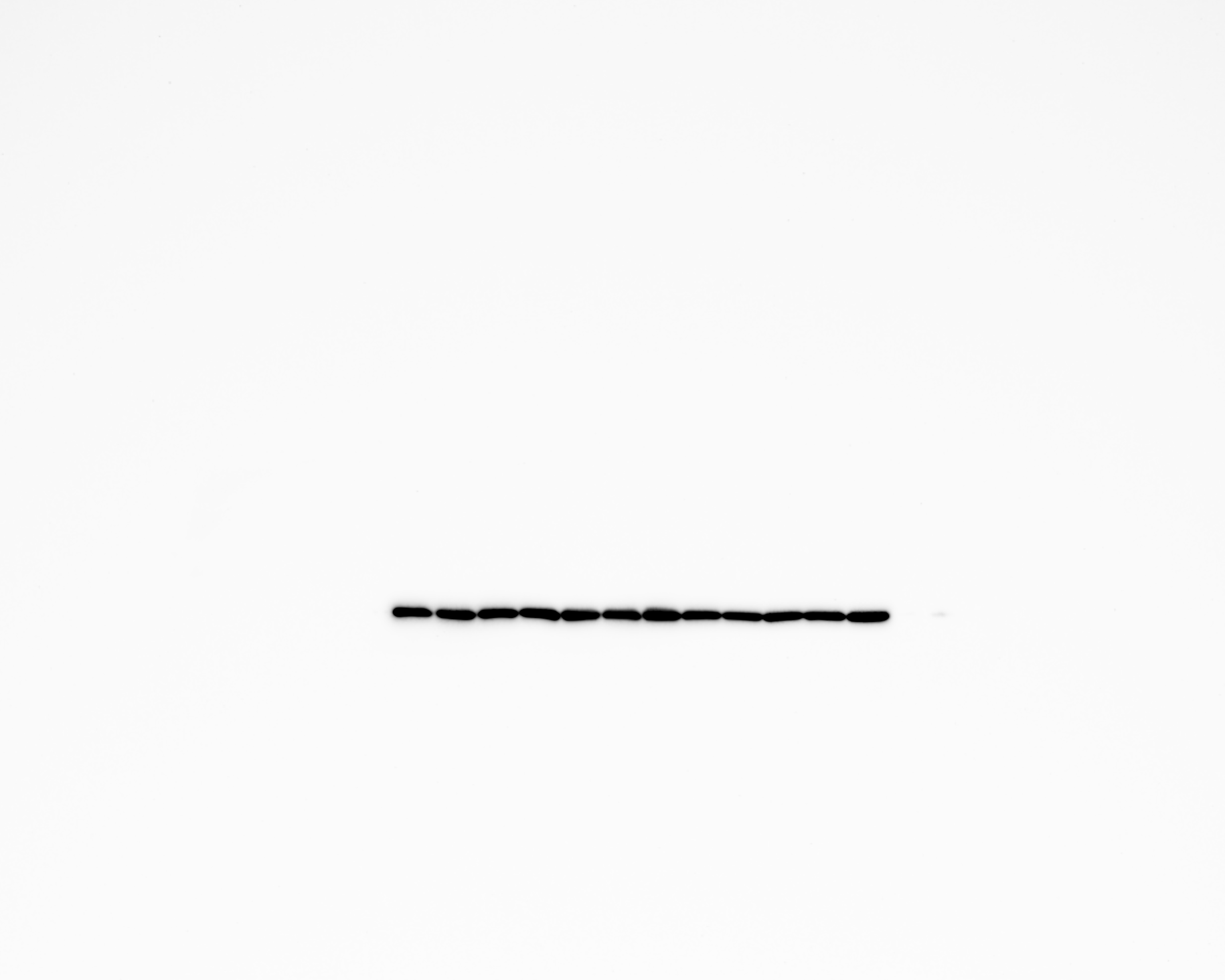

Supplement: Supplementary file 4 — Original immunoblot for supplementary figure 1 4-month beta actin [file 40478_2025_2162_MOESM4_ESM.tif]

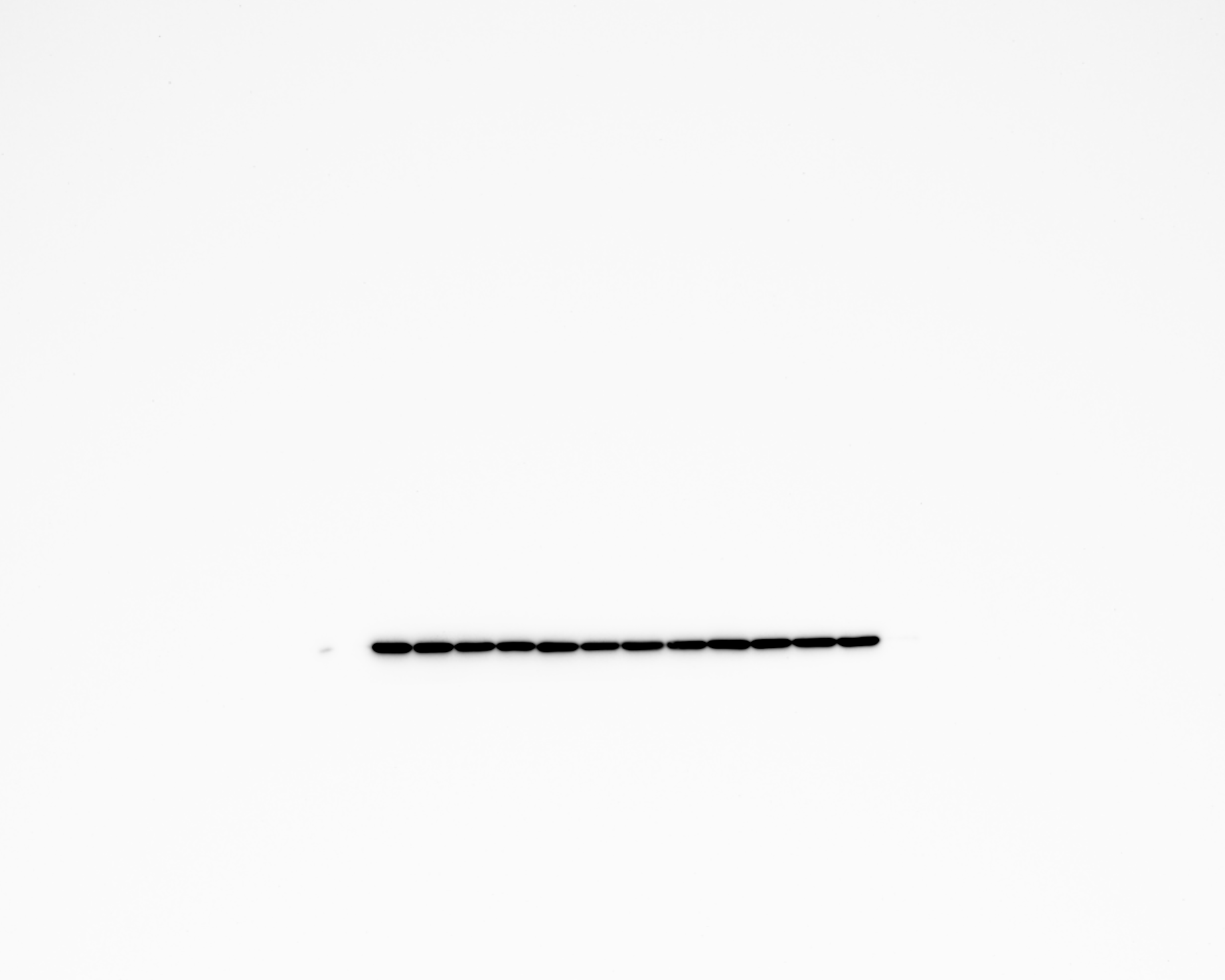

Supplement: Supplementary file 5 — Original immunoblot for supplementary figure 1 1-month beta actin [file 40478_2025_2162_MOESM5_ESM.tif]

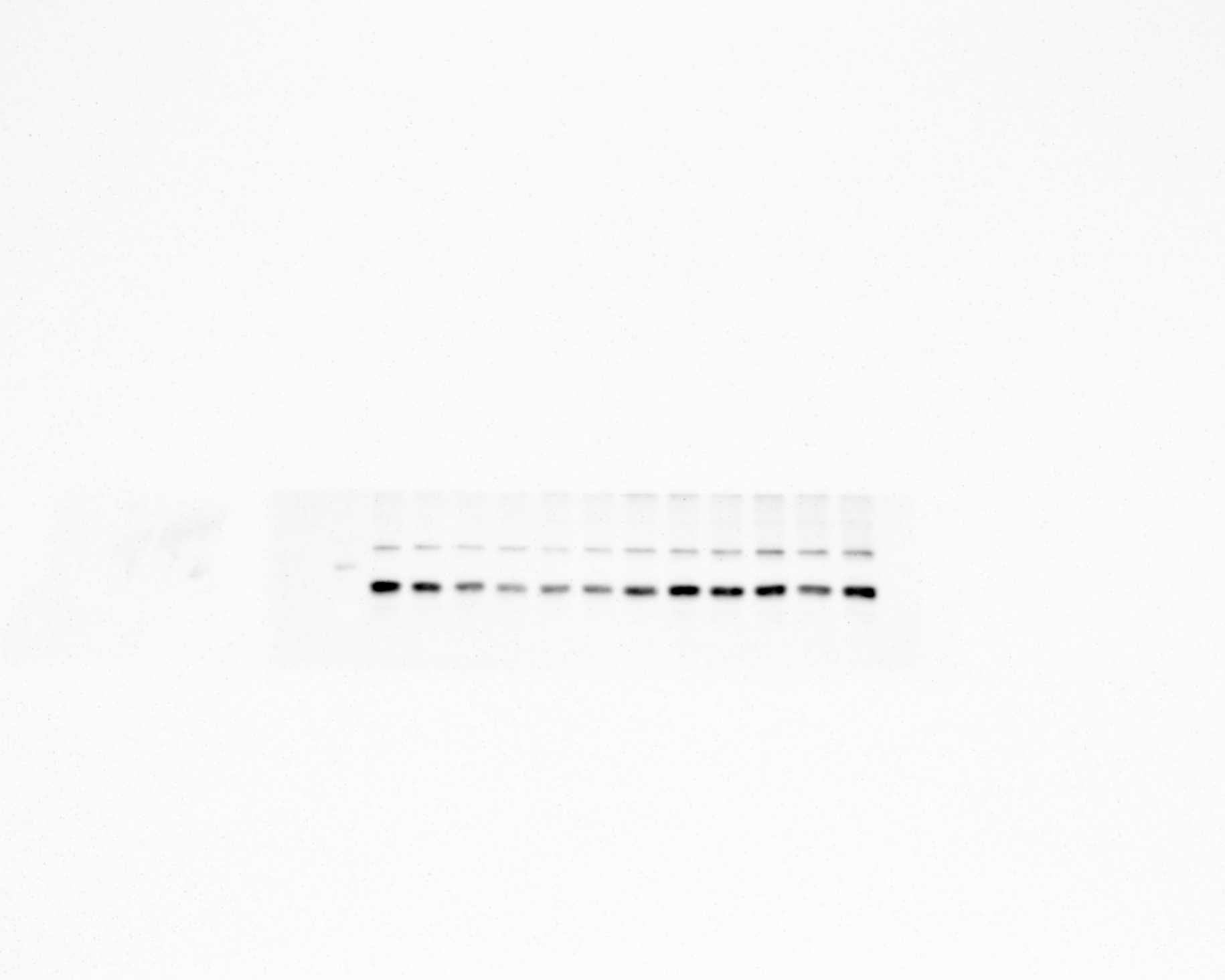

Supplement: Supplementary file 6 — Original immunoblot for supplementary figure 1 1-month MANF [file 40478_2025_2162_MOESM6_ESM.tif]

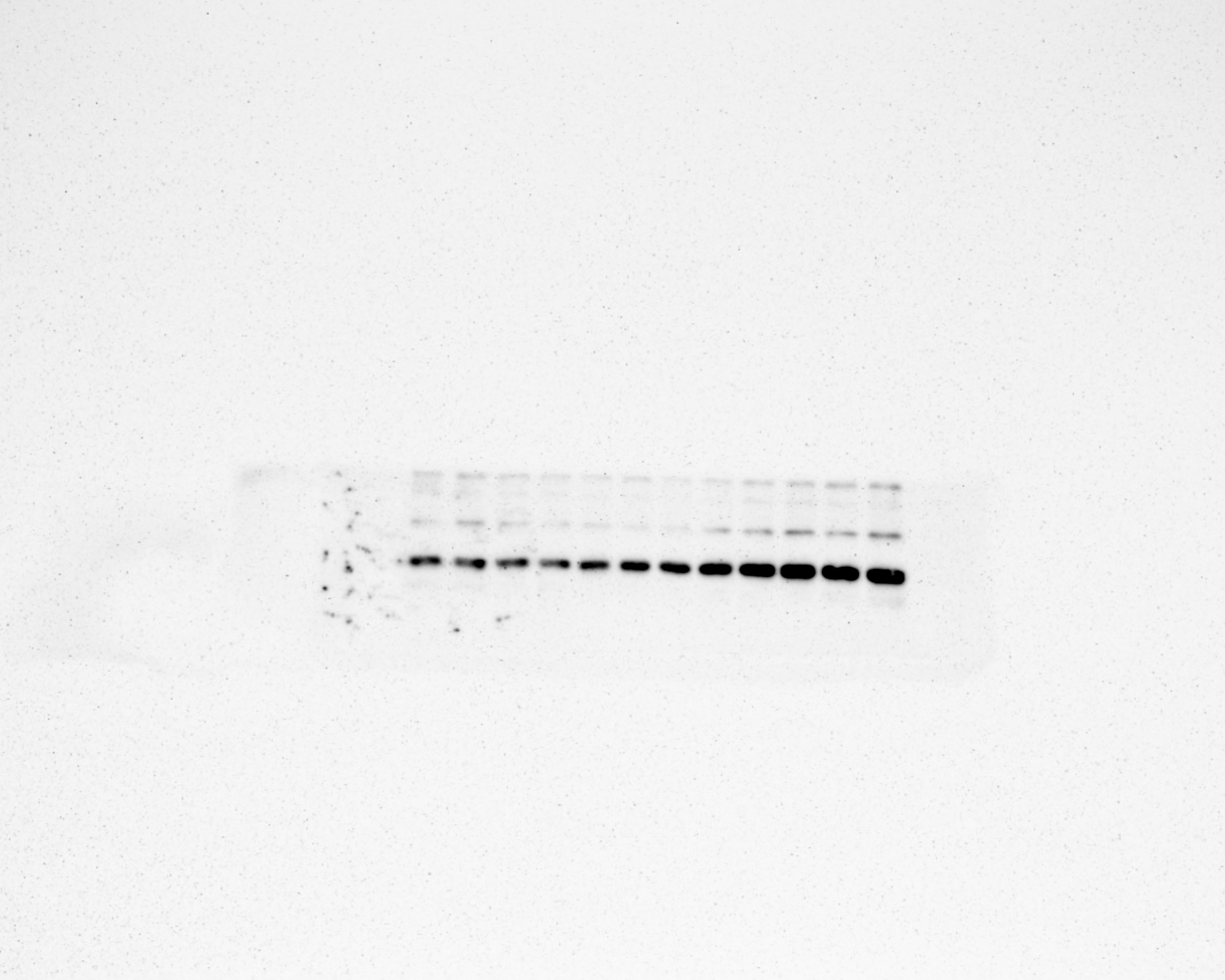

Supplement: Supplementary file 7 — Original immunoblot for supplementary figure 1 4-month MANF [file 40478_2025_2162_MOESM7_ESM.tif]
