# Supplementary material for: Spatial transcriptomics analysis uncovers ER stress in MANF-deficient Purkinje cells underlying alcohol-induced cerebellar neurodegeneration in mice
Source: Acta Neuropathol Commun. 2025 Dec 3;14:10. doi: 10.1186/s40478-025-02162-1 (PMC12781712; doi:10.1186/s40478-025-02162-1)
Supplement: Supplementary file 8 — Supplementary Figures [file 40478_2025_2162_MOESM8_ESM.docx]

**
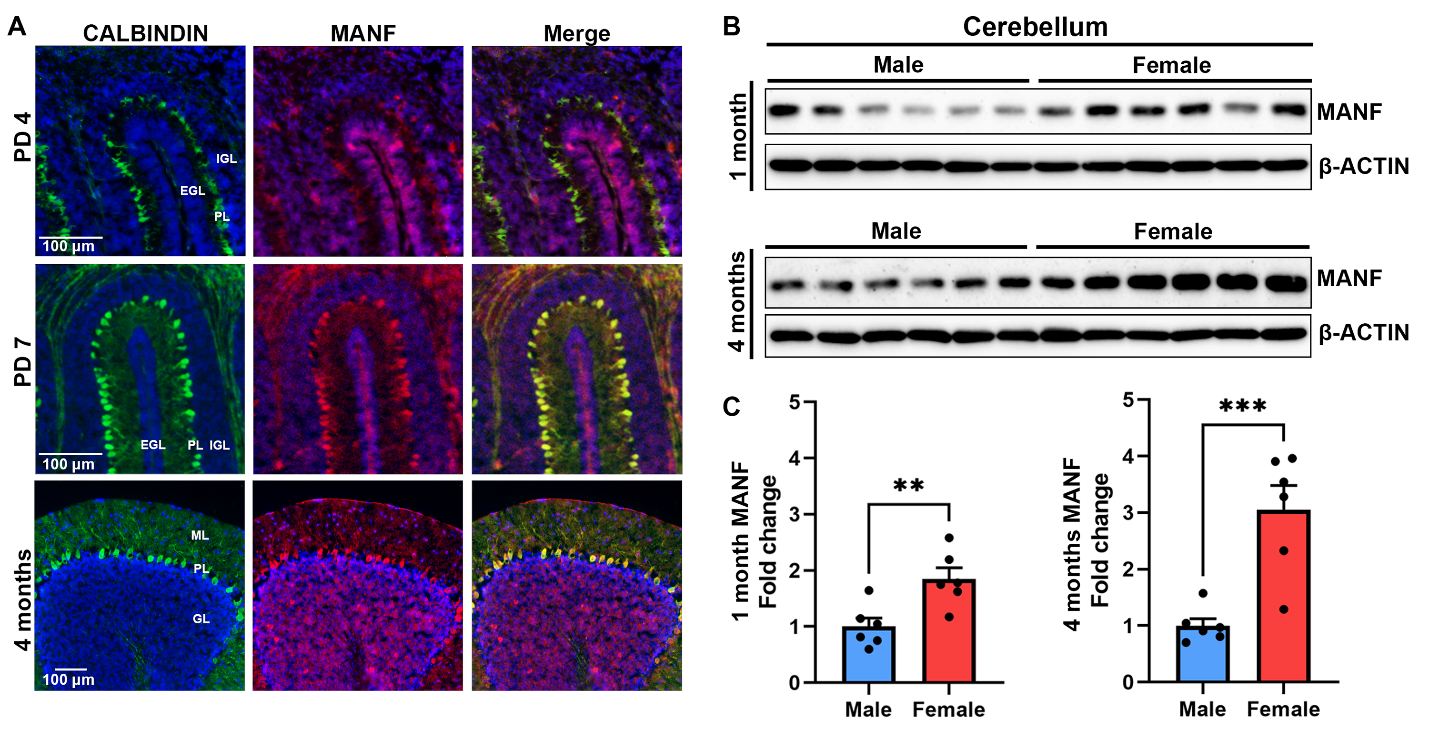
 Figure S1. MANF expression in C57BL/6 wild type mice cerebellum. A.** Representative immunofluorescent images of MANF (red) and PC marker CALBINDIN (green) in the C57BL/6 cerebellum at postnatal day (PD) 4, PD 7, and 4 months. Nuclei were counterstained with DAPI. EGL, external granular layer; IGL, internal granular layer; ML, molecular layer; PL, Purkinje cell layer; GL, granule cell layer. **B.** Representative immunoblot for MANF and β-ACTIN expression in 1- and 4-month-old C57BL/6 wild type male and female mice cerebellum. n=6 per group. **C.** Quantification of MANF expression normalized by β-ACTIN in 1- and 4-month C57BL/6 wild type male and female mice cerebellum. The data was expressed as mean ± SEM. n=6 per group. Student’s *t*-test. ***p*< 0.01; ****p*< 0.001.

**
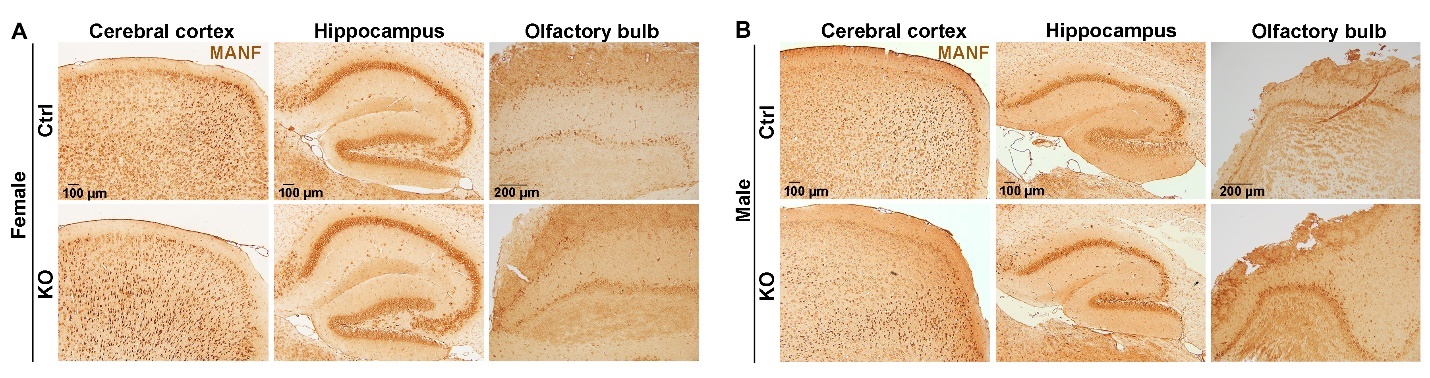
 Figure S2. MANF expression in other brain regions is not affected in PC-specific MANF KO animals. A-B.** Representative immunohistochemistry images showing comparable MANF expression in the cerebral cortex, hippocampus, and olfactory bulb of adult female (A) and male (B) control and KO mice.

**
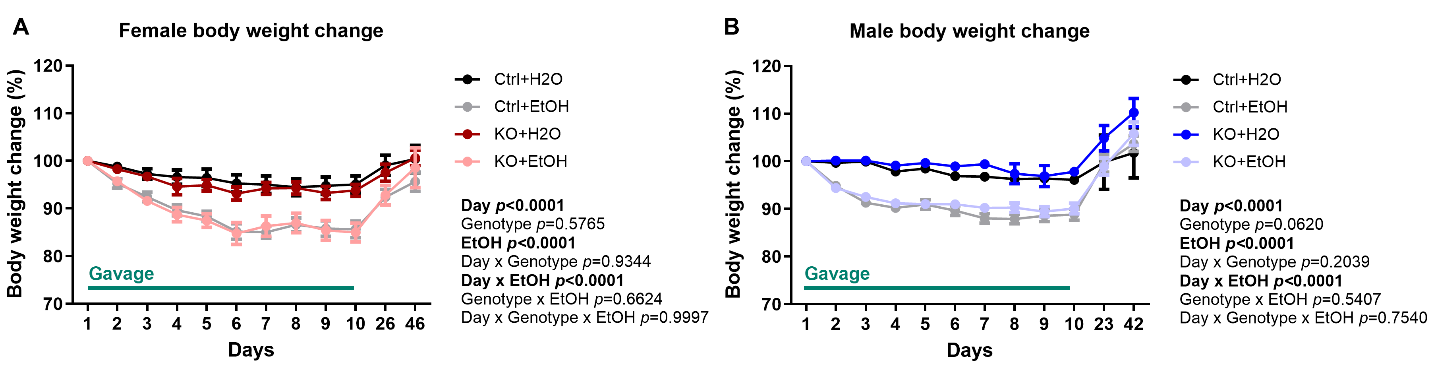
 Figure S3. Mice body weight change during gavage and behavior tests. A-B.** Body weight percentage change during gavage in female (A) and male (B). The data was expressed as mean ± SEM. n=8-11 per group. Three-way ANOVA. Significant main effect *p* values were highlighted in bold.


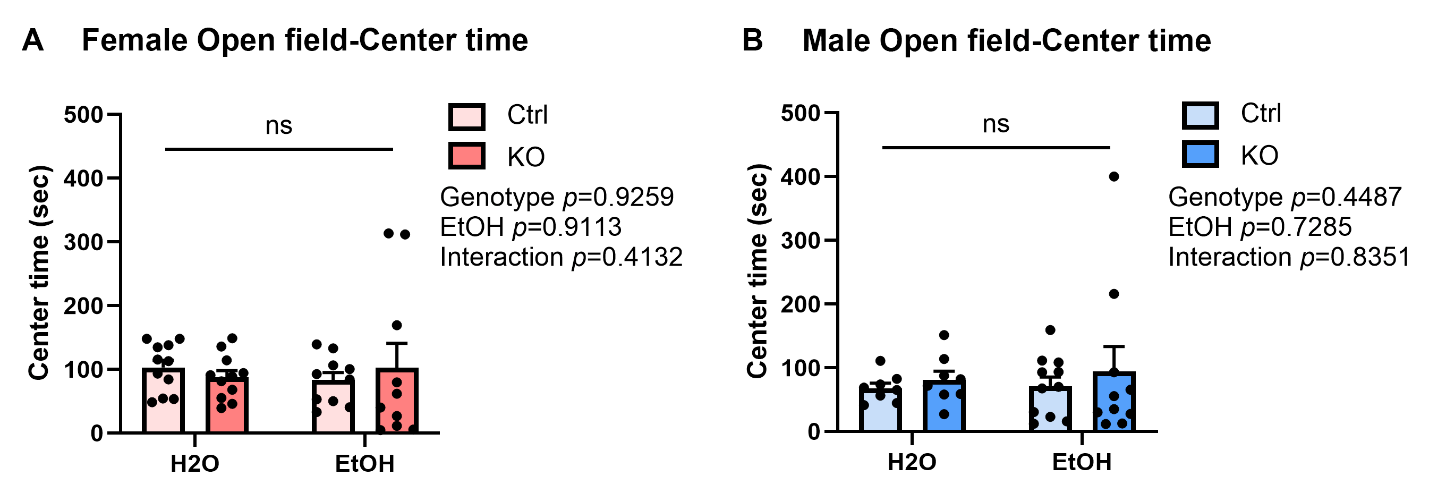


**Figure S4. Effects of ethanol exposure on anxiety-like behavior in open field test.** A-B. The time (sec) spent in the center of the open field arena in female (A) and male (B) mice. All data was expressed as mean ± SEM. n =8-11 per group. Two-way ANOVA followed by Tukey’s *post hoc* test. ns not significant.

**
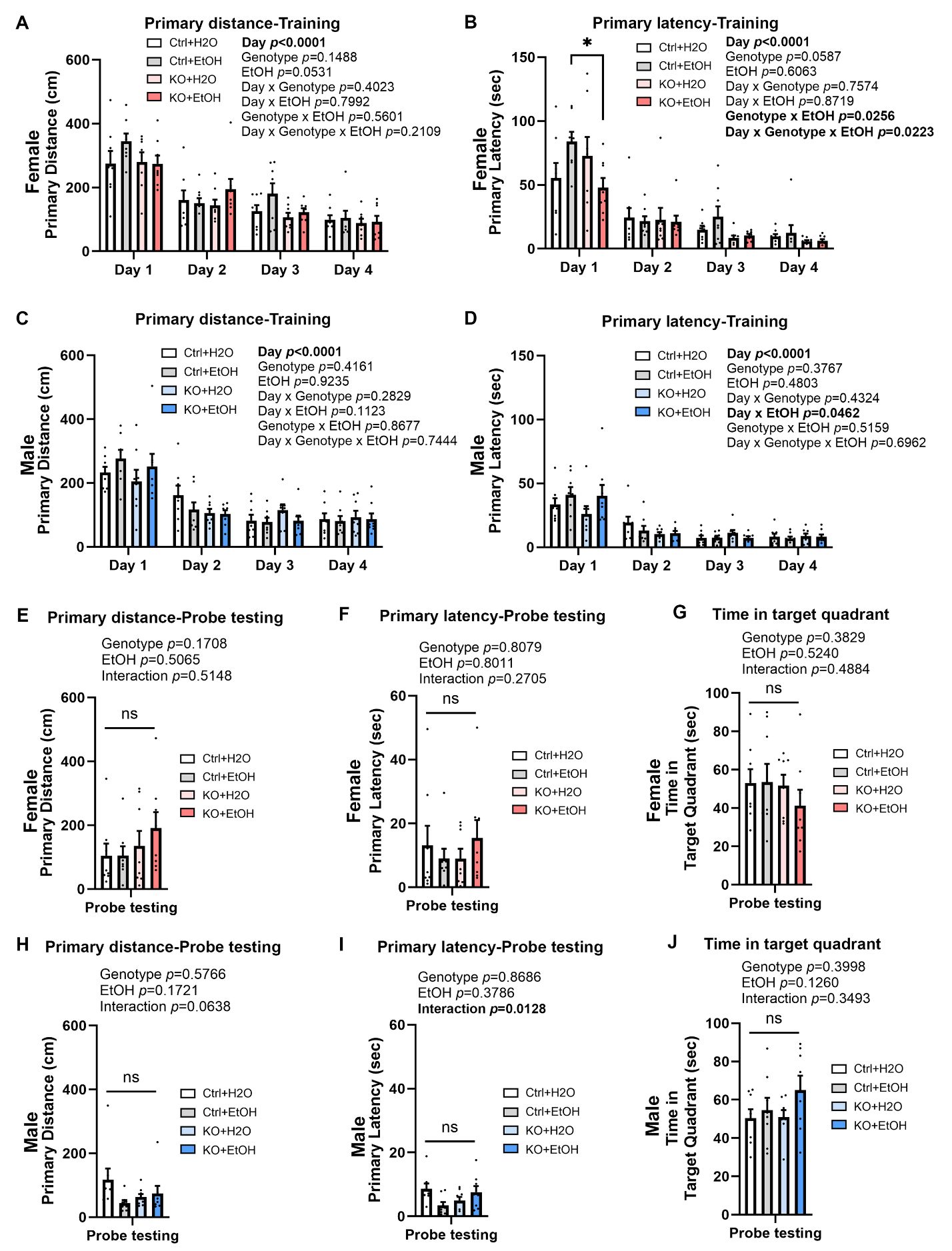
 Figure S5.** **Effects of ethanol exposure on learning and memory in Barnes maze test. A-D.** Primary distance traveled (A, C) and primary latency (B, D) to find the escape hole during training days in female (A, B) and male (C, D) mice. **E-J.** Primary distance traveled (E, H), primary latency (F, I) to find the escape hole, and time spent in the target quadrant (G, J) on probe testing day in female (E-G) and male (F-J) mice. All data was presented as mean ± SEM. n=8 per group. A-D, three-way ANOVA followed by Tukey’s *post hoc test*. E-J, two-way ANOVA followed by Tukey’s *post hoc* test. Significant main effect *p* values were highlighted in bold; **p*< 0.05; ns not significant.

**
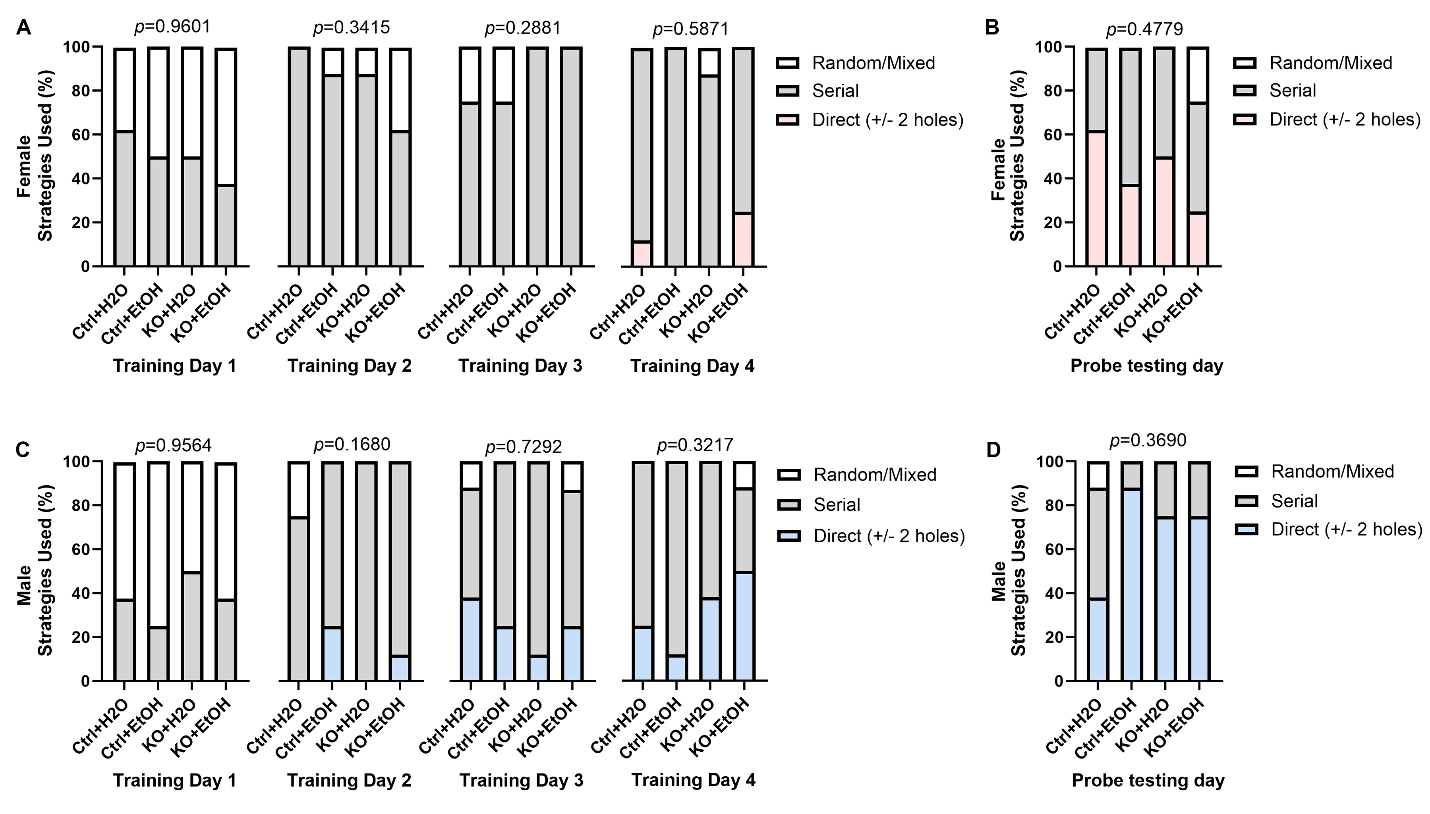
 Figure S6. Searching strategies used by control and MANF KO mice in Barnes maze test. A-B.** Percentage of each strategy used to find the escape hole during training (A) and probe testing period (B) in female. **C-D.** Percentage of each strategy used to find the escape hole during training (C) and probe testing period (D) in male. The three defined search strategies were random (top, white), serial (middle, gray), and direct (bottom, colored). n=8 per group. Fisher’s exact test. *p* values were listed on top of the bar graphs for each day.

**
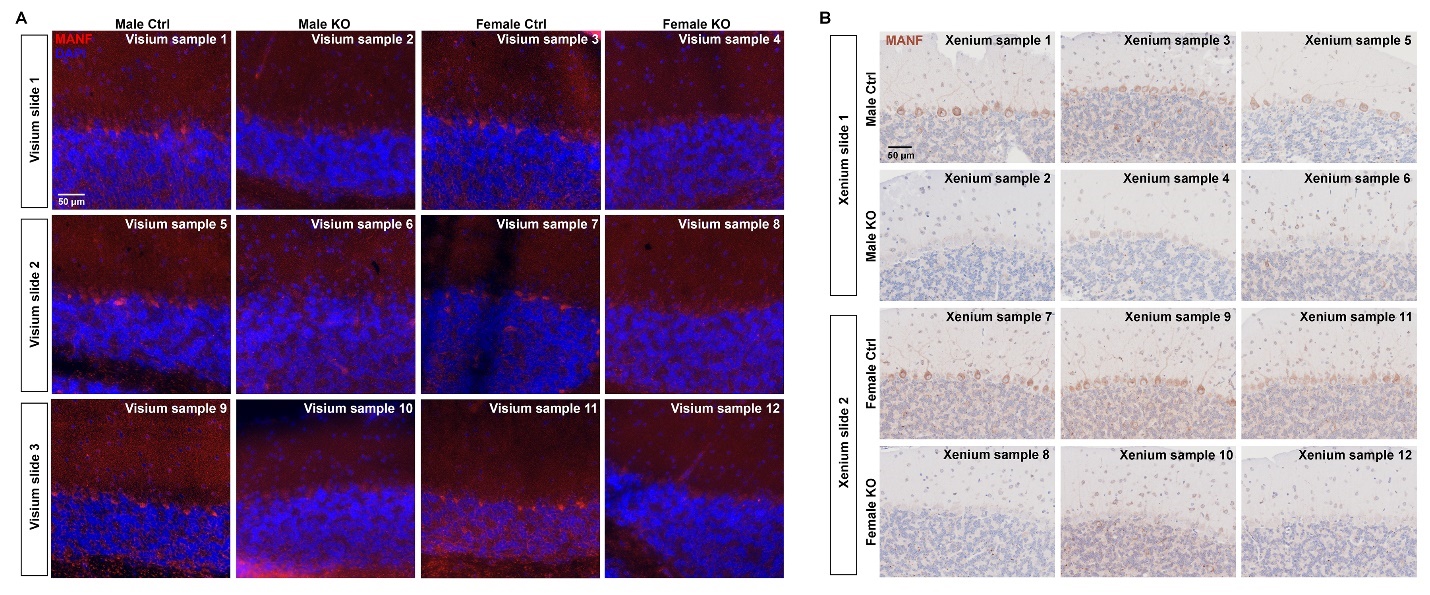
 Figure S7. MANF expression in Visium and Xenium samples. A.** Representative immunofluorescent images for MANF expression (red) in the 12 cerebellum samples used for Visium spatial transcriptomics analysis. Samples were counterstained with DAPI (blue). **B.** Representative immunohistochemistry images for MANF expression in the 12 cerebellum samples used for Xenium *in situ* analysis. Samples were counterstained with hematoxylin.
